# NIR diagnostic imaging of triple-negative breast cancer and its lymph node metastasis for high-efficiency hypoxia-activated multimodal therapy

**DOI:** 10.1186/s12951-023-02010-1

**Published:** 2023-09-02

**Authors:** Yi Pan, Longcai Liu, Yichen He, Luyi Ye, Xin Zhao, Zhiming Hu, Xiaozhou Mou, Yu Cai

**Affiliations:** 1 Center for Rehabilitation Medicine, Rehabilitation and Sports Medicine Research Institute of Zhejiang Province, Department of Rehabilitation Medicine, Cancer Center, Zhejiang Provincial People’s Hospital (Affiliated People’s Hospital), Hangzhou Medical College, Hangzhou, 310014 Zhejiang China; 2 College of Pharmacy, Zhejiang University of Technology, Hangzhou, 310014 Zhejiang China; 3 Clinical Research Institute, Zhejiang Provincial People’s Hospital (Affiliated People’s Hospital), Hangzhou Medical College, Hangzhou, 310014 Zhejiang China; 4 College of Pharmacy, Hangzhou Medical College, Hangzhou, 310059 China; 5 Department of Hepatobiliary Pancreatic Surgery, Zhejiang Provincial Tongde Hospital, Hangzhou, 310012 Zhejiang China

**Keywords:** Triple-negative breast cancer, Tumor hypoxia, Photodynamic therapy, Anti-angiogenesis, Hypoxia-activated therapy, Lymph node metastasis

## Abstract

**Background:**

Triple-negative breast cancer (TNBC) possesses special biological behavior and clinicopathological characteristics, which is highly invasive and propensity to metastasize to lymph nodes, leading to a worse prognosis than other types of breast cancer. Thus, the development of an effective therapeutic method is significant to improve the survival rate of TNBC patients.

**Results:**

In this work, a liposome-based theranostic nanosystem (ILA@Lip) was successfully prepared by simultaneously encapsulating IR 780 as the photosensitizer and lenvatinib as an anti-angiogenic agent, together with banoxantrone (AQ4N) molecule as the hypoxia-activated prodrug. The ILA@Lip can be applied for the near-infrared (NIR) fluorescence diagnostic imaging of TNBC and its lymph node metastasis for multimodal therapy. Lenvatinib in ILA@Lip can inhibit angiogenesis by cutting oxygen supply, thereby leading to enhanced hypoxia levels. Meanwhile, large amounts of reactive oxygen species (ROS) were produced while IR 780 was irradiated by an 808 nm laser, which also rapidly exhausted oxygen in tumor cells to worsen tumor hypoxia. Through creating an extremely hypoxic in TNBC, the conversion of non-toxic AQ4N to toxic AQ4 was much more efficiency for hypoxia-activated chemotherapy. Cytotoxicity assay of ILA@Lip indicated excellent biocompatibility with normal cells and tissues, but showed high toxicity in hypoxic breast cancer cells. Also, the in vivo tumors treated by the ILA@Lip with laser irradiation were admirably suppressed in both subcutaneous tumor model and orthotopic tumor models.

**Conclusion:**

Utilizing ILA@Lip is a profound strategy to create an extremely hypoxic tumor microenvironment for higher therapeutic efficacy of hypoxia-activated chemotherapy, which realized collective suppression of tumor growth and has promising potential for clinical translation.

**Supplementary Information:**

The online version contains supplementary material available at 10.1186/s12951-023-02010-1.

## Introduction

Triple-negative breast cancer (TNBC) refers to an aggressive subtype of breast case with negative results of estrogen receptor (ER), progesterone receptor (PR), and proto-oncogene Her-2 in immunohistochemical examination of cancer tissue [1, 2]. This type of breast cancer accounts for 10.0% ~ 20.8% of all pathological types of breast cancer that have special biological behavior and clinicopathological characteristics, higher frequency of metastasis, and the prognosis is worse than other types [3]. During the evolution of aggressive TNBC, early lymph node metastasis formated [4] and served as an important factors of the progression of breast cancer [5]. However, the lymph node metastasis of TNBC is difficult to diagnose [6]. Therefore, advanced TNBC is highly aggressive with a poor 5-year survival rate [7]. Currently, there are no specific treatment guidelines for TNBC [8, 9], although chemotherapy has a high response rate for TNBC, the prognosis is still poor as the standard routine [10, 11]. Therefore, it is of great significance to develop novel intelligent treatment methods for TNBC.

Hypoxia, as one of the typical features of TNBC, refers to the condition in which oxygen is insufficient to support the body’s metabolism [12, 13]. The hypoxia condition occurs when the vascular supply is interrupted or tumor growth exceeds its vascular supply [14, 15]. Because of the association with aggressive tumor phenotype and treatment resistance, hypoxia is a key factor for poor prognosis of cancer treatment [16]. Hypoxia is also a major cellular stress factor, which extensively affects multiple molecular metabolic pathways [17]. Based on these features, tumor hypoxia may be an attractive target for targeted therapy [18, 19]. Hypoxia-activated prodrugs (HAPs) have been proven to selectively kill hypoxic cancer cells, transforming hypoxia from a disadvantage to an advantage for precise treatment [20, 21]. As HAPs only contribute to cell-killing effects in hypoxic environments, thereby reducing side effects to normal tissue in normoxic conditions [22, 23]. However, insufficient hypoxia in tumor also severely limit the activation effectiveness and bioavailability of HAPs [24]. Therefore, finding ways to aggravate hypoxia may enhance the therapeutic effect of HAPs [25, 26].

Anti-angiogenic therapy (AAT) can inhibit the formation of tumor neovascularization factor VEGF through tumor vascular inhibitor [27], thereby inducing natural apoptosis of vascular endothelial cells [28] and destroying tumor neovascularization network [29]. AAT has been confirmed to cut off the oxygen and nutrition supply to the tumor [30], which enhance the degree of hypoxia inside the tumor [31]. Coincidentally, photodynamic therapy (PDT) specifically kills cancer cells in the presence of oxygen to generate reactive oxygen species (ROS) by photosensitizer under light irradiation [32], which also has the advantages of low systemic toxicity [33], low therapeutic resistance [34] and low invasiveness [35]. As PDT is highly oxygen-dependent, it can further lead to hypoxia in the tumor tissue [36, 37]. As an opposite strategy, the creation of a tumor hypoxic microenvironment using photosensitizer via PDT could be exploited for the activation of HAPs, contributing to a highly effective synergistic cancer therapy [38, 39]. In addition, the photosensitizer can be excited by the laser to exert photothermal therapy (PTT) effect for tumor elimination, which is independent of oxygen. Therefore, the combination of AAT, PDT and PTT is expected to significantly increase tumor hypoxia, thus extremely enhancing HAPs’ therapeutic effect.

**Scheme 1.**
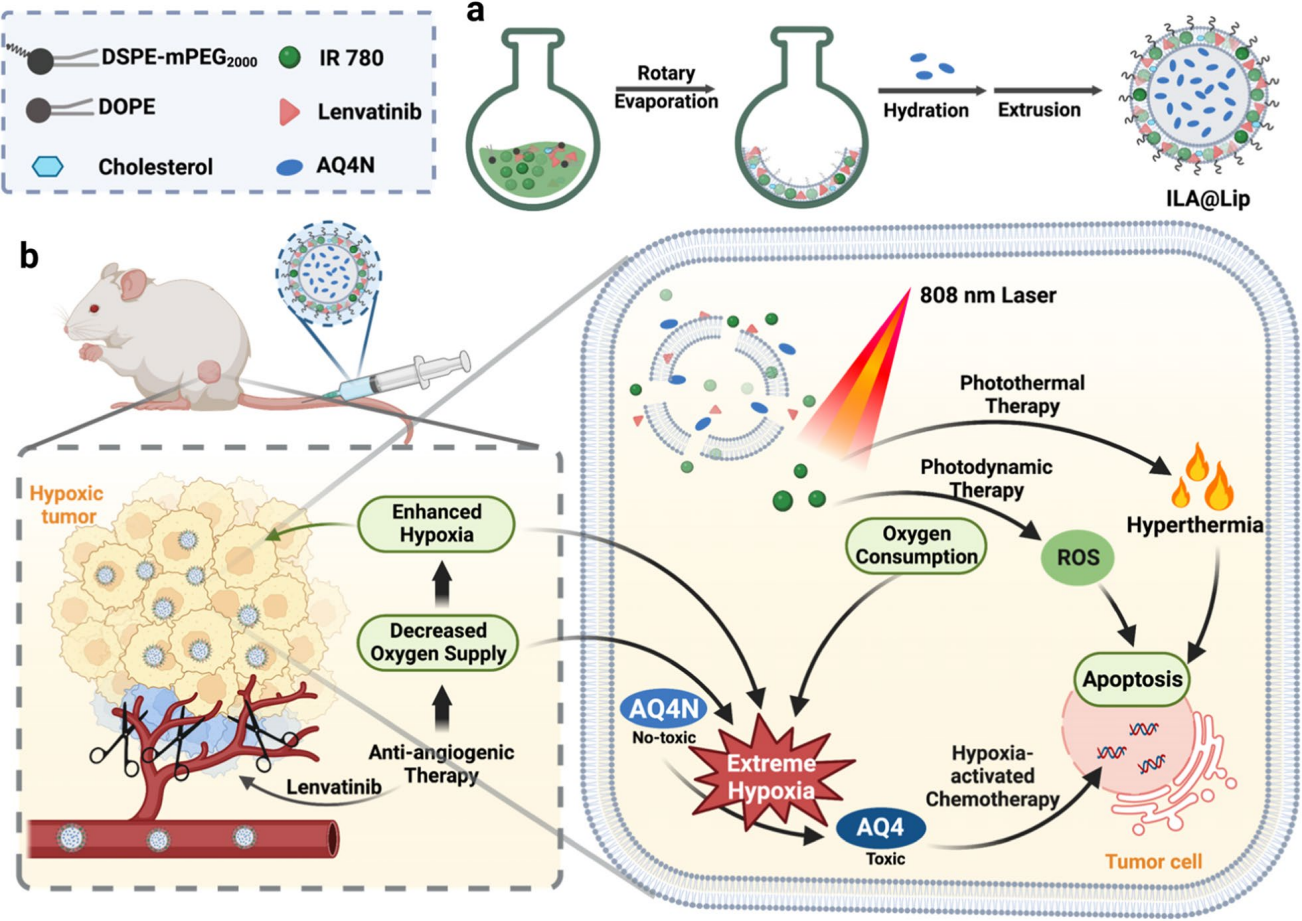
**a** Illustrations of the ILA@Lip preparation, and **b** synergistic effects in TNBC treatment via combining anti-angiogenic, photothermal therapy, photodynamic therapy, and hypoxia-activated chemotherapy

In this work, a liposome-based theranostic nanosystem (IR 780-Lenvatinib-AQ4N Lipsome, ILA@Lip) was prepared by simultaneously encapsulating hydrophobic IR 780 as the photosensitizer and hydrophobic lenvatinib as an anti-angiogenic agent into the lipid bilayer, together with banoxantrone (AQ4N) molecule as the hypoxia-activated prodrug into the hydrophilic core, respectively (Scheme 1a). The ILA@Lip can be applied for the near-infrared (NIR) fluorescence diagnostic imaging of TNBC and its lymph node metastasis for multimodal therapy. After accumulating in the TNBC tumor region, lenvatinib in ILA@Lip inhibited angiogenesis to cut oxygen supply, leading to enhanced hypoxia level. Besides, large amounts of ROS were produced with IR 780 under laser irradiation via consuming the remaining oxygen in tumor cells. As both pathways to reduce oxygen in tumor sites, aggravation of hypoxia led to the conversion of non-toxic AQ4N to toxic AQ4 for hypoxia-activated chemotherapy. It is demonstrated both in vitro and in vivo that the as-prepared ILA@Lip showed obvious anti-angiogenic ability, effective photodynamic cell-killing ability, photothermal therapy ability, and hypoxia-dependent cytotoxicity (Scheme 1b). By utilizing ILA@Lip, the synergistic effects of AAT and PDT were profound to create an extremely hypoxic tumor microenvironment (TME) to collectively benefit the therapeutic efficacy of hypoxia-activated chemotherapy and has promising potential for the clinical TNBC treatment.

## Experimental section

### Materials

1,2-Dioleoyl-sn-glycero3-phosphoethanolamine(DOPE), 1,2-distearoyl-sn-glycero-3-phosphoethanolamine-N-[methoxy(polyethyleneglycol)-2000] (DSPE-mPEG_2000_) were purchased from Avanti Polar Lipids Inc. (Alabaster, USA). Cholesterol was purchased from Sigma-Aldrich Co. (St. Louis, USA). IR 780 was purchased from Energy Chemical Co., Ltd (Shanghai, China). Lenvatinib was purchased from Aladdin Co., Ltd (Shanghai, China). Banoxantrone dihydrochloride (AQ4N) was provided by Abcam (Cambridge, UK). Cell counting kit-8 (CCK-8) was purchased from Yeasen Science & Technology Co., Ltd (Shanghai, China). Calcein/PI Cell Viability/Cytotoxicity Assay Kit, dichloro-dihydro-fluorescein diacetate (DCFH-DA), and Hoechst 33342 were purchased from Beyotime Biotechnology Co., Ltd (Shanghai, China). Matrigel was purchased from R and D systems. The primary antibodies for hypoxia-induced factor alpha (HIF-1α) and secondary antibodies were purchased from Cell Signaling Technology (USA). Fetal bovine serum (FBS) and Dulbecco's Modified Eagle medium (DMEM) were purchased from Biological Industrie (Shanghai, China). All other chemical reagents were analytically pure and used directly as received.

### Preparation of ILA@Lip

To preparation procedure of ILA@Lip is as follows: the lipid mixture of 11.54 mg DOPE, 5.62 mg DSPE-mPEG_2000_, 2 mg cholesterol, 1 mg IR 780, and 1 mg lenvatinib were dissolved in 12 mL chloroform and 4.5 mL methanol. Then the solution was dried under a rotary evaporator at 80 r min^−1^ and 35 °C for 3 h to obtain a thin lipid film in a round bottom flask. Afterward, the dried lipid film was hydrated with an AQ4N solution (10 mg mL^−1^) and stirred at 45 °C for 30 min. Then, the suspension was extruded through a polycarbonate membrane of 200 nm using an extruder (Avanti Polar Lipids Inc). The obtained liposome, named ILA@Lip.

### Characterization

The morphology of ILA@Lip was examined by transmission electron microscope (TEM, Hitachi H-7650). The size distribution (diameter, nm) and surface charge (zeta potential, mV) of the nanoparticles were measured by the Zetasizer Nano ZS particle analyzer (Malvern Instruments Limited). The UV–vis absorption spectrums of nanoparticles were recorded using a UV–vis spectrophotometer (Thermo Fisher). The concentrations of IR 780 and AQ4N in the liposome were determined using a UV–vis absorbance spectrometer. The UV–Vis spectra of IR 780 ethanol solution with various standard concentrations were measured via the UV–Vis spectrophotometer. The absorbances at 784 nm were recorded to construct a standard concentration curve for determining the concentration of IR 780. In vitro stability of ILA@Lip in Phosphate buffered saline (PBS) was monitored by Zetasizer Nano ZS particle analyzer and UV–vis spectrophotometer on the 0 day, 7 day, and 14 day after the liposome was synthesized. The heat generated by the laser was monitored by a thermal imager camera (FLUKE, TI400). The encapsulation efficiency of the reagent was determined as follows: isolating the fresh liposomes from aqueous suspension medium by ultracentrifuge (20000 rpm, 60 min). The supernatant containing the free drugs was removed. Then the concentration of IR 780 was determined by a UV–vis spectrophotometer The encapsulation efficiency and drug loading content of the reagents in ILA@Lip were calculated using the following formulas:

$$ {\text{Encapsulation efficiency }}\left( {{\text{wt\% }}} \right) = \frac{{{\text{mass}}\,{\text{of}}\,{\text{the}}\,{\text{reagent}}\,{\text{in}}\,{\text{liposome}}}}{{{\text{mass}}\,{\text{of}}\,{\text{the}}\,{\text{reagent\,in}}\,{\text{feed}}}} \times 100\% $$

$$ {\text{Loading content }}\left( {{\text{wt}}\% } \right) = \frac{{{\text{mass}}{\mkern 1mu} \,{\text{of}}\,{\mkern 1mu} {\text{the}}{\mkern 1mu} \,{\text{reagent}}\,{\mkern 1mu} {\text{in}}{\mkern 1mu} \,{\text{liposome}}}}{{{\text{the}}{\mkern 1mu} \,{\text{total}}{\mkern 1mu} \,{\text{mass}}{\mkern 1mu} \,{\text{of}}{\mkern 1mu} \,{\text{the}}\,{\mkern 1mu} {\text{liposome}}}} \times 100\%  $$

### Cell culture

4T1 cells (mouse breast carcinoma) and MDA-MB-231 (human breast carcinoma) cells were cultured in DMEM with 21% O_2_ and 5% CO_2_ at 37 °C. HUVEC cells and L929 cells were cultured in DMEM with 21% O_2_ and 5% CO_2_ at 37 °C. To mimic the hypoxia tumor microenvironment, the hypoxic cells were maintained at 1% O_2_ and 5% CO_2_ by Anaero Park TM-MicroAero (Mitsubishi, Japan) at 37 °C. All culture media contained 10% fetal bovine serum (Biological Industries), 1% penicillin, and 1% streptomycin (New cell & Molecular Biotech).

### In vitro cellular uptake

The cellular uptake of ILA@Lip in MDA-MB-231 cells was examined using confocal microscopy(LEICA DMI8). Free IR 780 and AQ4N were used for comparison. Briefly, MDA-MB-231 cells (1 × 10^5^ cells) were seeded into coverglass bottom dishes in 1 mL medium. After 24 h, the medium was replaced with the medium containing free IR 780 (2 μg mL^−1^), AQ4N (2 μg mL^−1^), or ILA@Lip (2 μg mL^−1^ IR 780 and 2 μg mL^−1^ AQ4N). After 2 h or 6 h incubation, the cells were washed thrice with PBS and stained with Hoechst 33342. These cells were observed by confocal microscope (LEICA DMI8) with fixed excitation wavelength. Quantitative results were investigated by flow cytometry analysis and the data were analyzed using Flow Jo software.

To evaluate the cellular uptake efficacy of ILA@Lip under hypoxic condition, MDA-MB-231 cells pre-seeded in a coverglass bottom dishes at a density of 1 × 10^5^ cells per dish were incubated with the medium containing ILA@Lip in a hypoxia incubator (1% O_2_, 5% CO_2_). After then, the cells were washed with PBS and stained with Hoechst 33342 for 20 min before being observed using a confocal laser scanning microscope.

### In vitro cytotoxicity assay

The viability of MDA-MB-231 cells treated with ILA@Lip with laser and without laser was assessed by CCK-8 (Yeasen Biotechnology, China). Briefly, 1 × 10^4^ cells were seeded in 96-well plates and incubated in 5% CO_2_ at 37 °C until adherence. Then the culture medium was removed and replaced with a series of concentrations of ILA@Lip diluted by DMEM medium. After 8 h incubation, cells were washed twice and cultured in fresh medium. The cells were irradiated with 808 nm laser (1.0 W cm^−2^) for 5 min in the “with laser” groups. After another 4 h of incubation at 37 °C. Subsequently, cell viability was measured through CCK-8 (Yeasen Biotechnology, China) detection according to the manufacturer’s protocol. Untreated cells were used as controls, corresponding to 100% of cell viability. The cytotoxicity of ILA@Lip on HUVEC and L929 cells was evaluated by CCK-8 as a similar procedure.

To test the cytotoxicity of ILA@Lip under hypoxic conditions, pre-seeded MDA-MB-231 cells (1 × 10^4^ cells per well) were incubated with a series concentration of ILA@Lip for 8 h under hypoxic condition (1% O_2_, 5% CO_2_). After that, these cells were re-cultured with the fresh medium, exposed to an 808 nm laser (1.0 W cm^−2^) for 5 min, and incubated for another 4 h under hypoxic condition before their cell viability being quantified via the CCK-8 assay.

Live/Dead cell viability/cytotoxicity assay was performed in MDA-MB-231 to qualitatively evaluate cell viability. In this assay, calcein-AM can be enzymatically converted into green fluorescent calcein in live cells, while propidium iodide(PI) stains the nuclei of dead cells with red fluorescence. MDA-MB-231 cells were incubated in 24-well plates until adherence. Fresh DMEM containing different substances (medium only, AQ4N, Lenvatinib, IR 780, ILA@Lip) was added followed by incubation for 8 h. Then, the cells in the IR 780 group and ILA@LIP group were exposed to 808 nm laser irradiation(1.0 W cm^−2^) for 5 min. Then the medium was replaced with 1 mL PBS containing calcien-AM and PI, to stain live and dead cells. Then, the cells were monitored with a microscope. In addition, the therapeutic effect in vitro was also evaluated by flow cytometry (stained with Annexin V-FITC and PI). To evaluate the effect of hypoxia on the toxicity, pre-seeded MDA-MB-231 cells were incubated under hypoxic condition (1% O_2_, 5% CO_2_). Other conditions were kept consistent to carry out the live-dead staining experiments and flow cytometry as described above.

### Detection of ^***1***^***O***_***2***_ generation

The 1,3-diphenylisobenzofuran (DPBF) is an indicator of ^1^O_2_, which could irreversibly react with ^1^O_2_ to decrease the UV absorbance. The DPBF was dissolved in 20 μL methanol and mixed with 1 mL ILA@Lip. The mixed solution was irradiated with a laser at 808 nm (1.0 W cm^−2^) and measured with a UV–vis spectrophotometer at an excitation wavelength of 494 nm.

The solutions of ILA@Lip in PBS were mixed with a commercial singlet oxygen sensor green (SOSG) probe at a final concentration of 2.5 μM and then subjected to a 808 nm laser. The fluorescence intensity of SOSG was recorded after a series of periods of irradiation.

### Analysis of intracellular ROS generation

The ROS production of ILA@Lip inside MDA-MB-231 cells was detected by DCFH-DA assay. Briefly, MDA-MB-231 cells were seeded in coverglass bottom dishes at a density of 1 × 10^5^ cells per dish and incubated for 24 h. Fresh DMEM containing different substances (medium only, AQ4N, lenvatinib, IR 780, and ILA@Lip) replaced the old medium and further incubated for 4 h. Then the medium was replaced with a fresh medium containing DCFH-DA (Beyotime Biotechnology, China) and incubated at 37 °C for 30 min before treating with/without laser irradiation (808 nm, 5 min, 1.0 W cm^−2^). Subsequently, the cells of the diverse groups were stained with Hoechst 33342 for 30 min and investigated by confocal microscopy (LEICA DMI8). For flow cytometry analysis, MDA-MB-231 cells were seeded into six-well plates at a density of 3 × 10^5^ cells per well and cultured for 24 h. Then, the cells were treated with different substances at an identical concentration of IR 780(5 μg mL^−1^). After 8 h of incubation, the cells were stained with DCFH-DA for 30 min and then exposed to an 808 nm laser for 5 min (1.0 W cm^−2^). These cells were harvested, and ROS generation was determined by flow cytometry analysis.

### Tube formation and tube broken assay

Tube-like structure broken assay of HUVECs was conducted on Matrigel (R and D systems). Matrigel was unfrozen at 4 °C overnight, spread evenly over each well (200 μL) of 24-well plates, and polymerized for 30 min at 37 °C. HUVECs (6 × 10^4^ cells/well) were plated onto the matrigel layer and cultured at 37 °C until tube formation could be observed. Then different mediums containing lenvatinib, AQ4N, IR 780, or ILA@Lip were added into the wells at the concentration. And in IR 780 and ILA@Lip group, 808 nm laser (1.0 W cm^–2^) irradiation was performed. As a result, the tube-like structures broke up 5 h after incubation. Pictures of broken tubes were captured with inverted microscopy after 5 h of incubation and 10 h of incubation.

HUVECs’ capacity of tube formation on Matrigel (R and D systems) after diverse treatments was tested. HUVECs pretreated with lenvatinib, AQ4N, IR 780, or ILA@Lip with or without laser irradiation. Then the trypsin-digested cell suspensions of HUVECs were spread on polymerized Matrigel. The tube formation efficiency was monitored after 5 h using the microscope. ImageJ software was used to calculate the number of junctions and length of tubes.

### Animals and tumor animal model establishment

All experimental animal procedures were performed with approval from the Institutional Animal Care and Use Committee of the Hospital of Zhejiang Province (GB/T 35892–2018). Female BALB/c mice (6–8 weeks old) were purchased from Slaccas Laboratory Animal Co., Ltd. (Shanghai, China). All mice had free access to food and water in standard conditions. To develop subcutaneous tumors, 1 × 10^6^ 4T1 cells suspended in 100 μL PBS were subcutaneously injected into the back of female BALB/c mice. When the tumor volume reached ~ 100 mm^3^, mice were randomly allocated to diverse groups for subsequent experiments. To create an orthotopic 4T1 breast tumor-bearing mouse model, 1 × 10^6^ 4T1 cells suspended in 100 μL PBS were injected into the fourth breast fat pad of female nude mice. When the tumor volume reached ~ 100 mm^3^, mice were randomly allocated to diverse groups for subsequent experiments. Tumor size and mouse weight were recorded every other day. The tumor volume was calculated using the formula: length × width^2^ × 0.5.

### Tumor accumulation in vivo

In vivo fluorescence imaging was initially used to evaluate the biodistribution and the moment of maximum drug enrichment within the tumor on subcutaneous and orthotopic 4T1 models. After seven days of feeding, the 4T1 tumor-bearing mice were injected with ILA@Lip. The fluorescence imaging of mice was conducted at determined time points after injection using an IVIS imaging system. After 48 h, the mice were sacrificed, and the heart, liver, spleen, lung, kidney, and tumor were taken for fluorescent imaging and analysis.

### In vivo antitumor efficacy and function

Subcutaneous 4T1 tumors in the right back were established as described above, and the treatment commenced when the tumor volume reached ~ 100 mm^3^. The mice in the diverse group were intravenously injected with PBS, Lenvatinib, AQ4N, IR 780, and ILA@Lip via tail. Tumors in the IR 780 or ILA@Lip plus irradiation group were exposed to 808 laser (1.0 W cm^−2^, 10 min) irradiation at 24 h after injection. These treatments were administered every 2 days for 3 cycles. The tumor size and body weight of each mouse were measured every other day. At the end of the treatment, all the mice were sacrificed. Major organs, including the lung, heart, kidney, spleen, and liver were removed for hematoxylin and eosin (H and E) staining and photographed to evaluate the safety of various treatments. To investigate the toxicity of diverse treatments in vivo, the sera of tumor-bearing mice were attained to estimate liver function (aspartate aminotransferase (AST), alanine aminotransferase (ALT)) and renal function (blood urea nitrogen (BUN), and creatinine (CR)) at the end of treatment. Furthermore, tumor tissues were harvested for H and E, and Ki-67 antibody staining for histological analysis. The tumor sections were stained with anti-CD31 antibody to label the tumor vascular. To investigate the level of hypoxia in vivo, tumors obtained from mice were stained with HIF-1α. For semiquantification, three or more fields of view were taken as fluorescent images for each group. The fluorescent intensity in each image was semiquantitated with Image J and averaged. Results were displayed as mean fluorescence intensity.

### TNBC lymphatic metastasis imaging and surgical resection

The 4T1-Luc cells in the logarithmic phase of growth were collected, washed once with PBS solution, diluted to 1 × 10^6^ mL^−1^ with PBS solution, and 50 μL of the cell suspension was slowly injected into the subcutaneous paw pad of the unilateral hind limb of BALB/c nude mice with a syringe to produce a mouse breast cancer paw pad subcutaneous lymphatic metastasis model. After two weeks, mice were injected intraperitoneally with sodium fluorescein and then performed bioluminescence imaging, which verifying the successful establishment of lymph node metastasis tumor models.

In a lymph node metastasis mouse model, ILA@Lip (AQ4N: 5 mg kg^−1^, Lenvatinib: 100 mg kg^−1^, IR 780: 5 mg kg^−1^) was injected via the tail vein, and fluorescence imaging was performed. Surgical resection of the tumor at the lymph nodes was performed under fluorescence imaging guidance. The procedure was photographed and recorded. The popliteal lymph nodes were dissected under aseptic conditions and the specimens were fixed in 4% paraformaldehyde. After fixation, the specimens were sectioned, HE stained, and pathological histological examination was performed to evaluate the metastasis of tumor cells.

### Statistics

All statistical analyses were conducted using GraphPad Prism 9.0.0 Software. The results are expressed as mean ± SD. Data sample size and probability values were indicated in figure legends. Nonparametric two-tailed analysis of variance (ANOVA) followed by a Tukey post-hoc test was used for multiple-group comparisons. P values ≤ 0.05 were considered statistically significant.

## Results and discussion

### Preparation and characterization of ILA@Lip

**Fig. 1:**
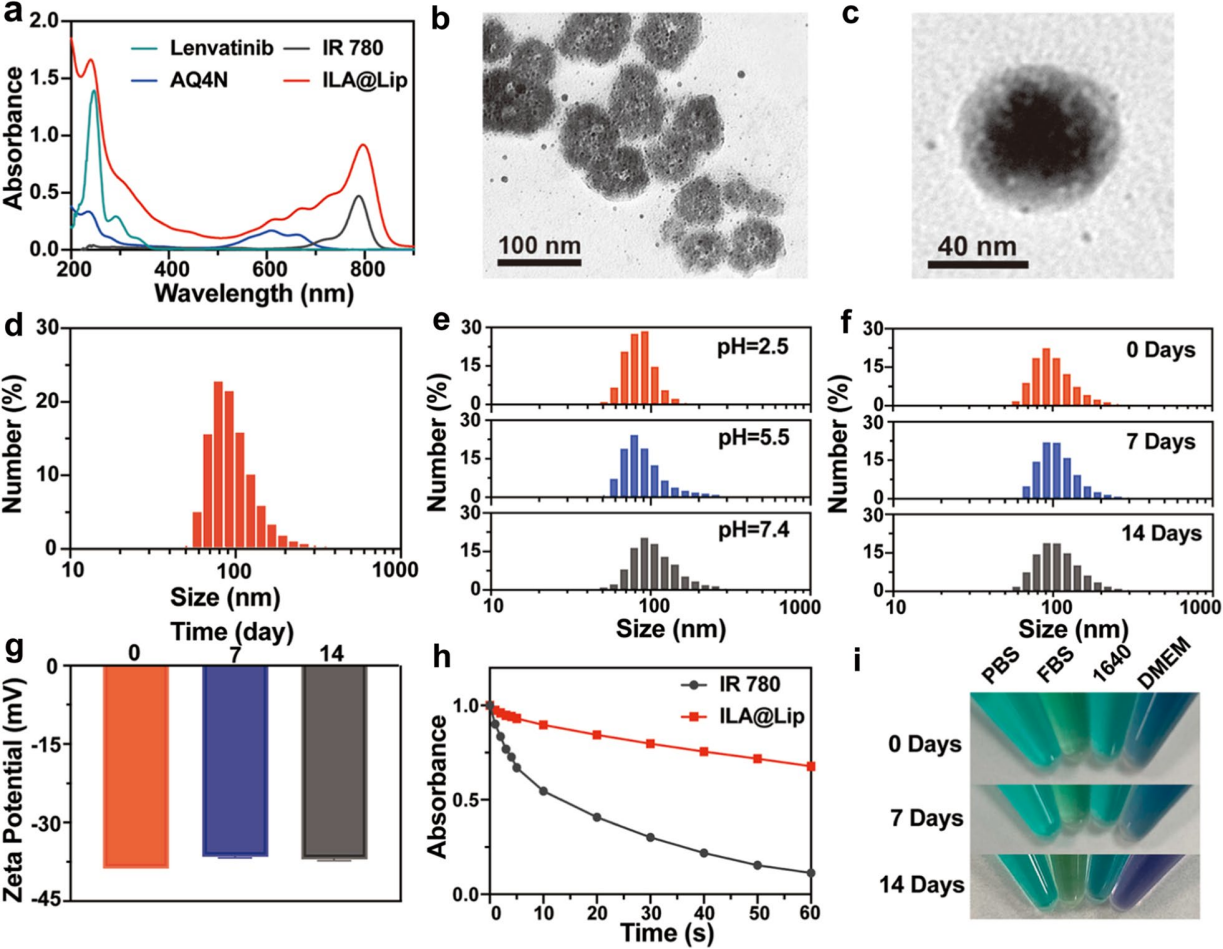
Characterization of ILA@Lip. **a** UV–vis–NIR absorption spectrum of lenvatinib, AQ4N, IR 780, ILA@Lip. **b** TEM image of ILA@Lip in water solution. Scale bar = 100 nm. **c** TEM image of an enlarged detail of ILA@Lip. **d**. The particle size distribution of ILA@Lip. Mean only, n = 3. **e** Relative particle size distribution of ILA@Lip in PBS of different pH. **f** Relative particle size distribution changes of ILA@Lip after the store for 0 days, 7 days, and 14 days. Mean only, n = 3. **g** The surface zeta potential of ILA@Lip after the store for 0 days, 7 days, and 14 days. Mean ± S.D., n = 3. **h** Absorbance decay curves of free IR 780, and ILA@Lip at 780 nm after the radiation of 808 nm laser (1.0 W cm^−2^) for a period of time. Results are presented as percentages by normalizing to absorbance intensity obtained before laser irradiation. Mean only, n = 3. **i** The corresponding photographs of ILA@Lip were stored in PBS, FBS, RPMI 1640, and DMEM for 0 days, 7 days, and 14 days

In consideration of both hydrophobic and hydrophilic properties of these utilized reagents being loaded in one system, the high tumor accumulation and excellent biocompatibility liposome was selected as the drug carrier [40]. The preparation of ILA@Lip is illustrated in Scheme 1a via a typical thin-film spin evaporation process. In brief, ILA@Lip was prepared by hydrating the dried lipid films formed by mixing DOPE, DSPE-mPEG_2000_, cholesterol, IR 780 and lenvatinib with PBS containing AQ4N according to the standard method for the preparation of liposomes [41]. Due to the difference in solubility, the hydrophobic lenvatinib and IR 780 was encapsulated into the lipid bilayer of the liposomes and the hydrophilic AQ4N existed in the core of ILA@Lip. The presence of IR 780 in the lipid bilayer made it easy to destruct the shells after laser irradiation to release encapsulated reagents without thermal inactivation. The successful encapsulation of lenvatinib, IR 780, and AQ4N into ILA@Lip was firstly confirmed by their characteristic absorbance peaks on the UV–vis absorbance spectrum (Fig. 1a). As expected, the absorption peak at 610 nm belongs to the characteristic absorption of AQ4N, and another peak at 240 nm belongs to the absorption of lenvatinib, respectively. The absorption spectra showed the ILA@Lip had a strong absorption peak at 799 nm, reflecting a redshift of characteristic absorption of free IR 780 at 780 nm. Such a strong absorption of ILA@Lip in the NIR region indicated a potential for PTT upon laser irradiation. The loading ratio among these drugs is designed according to the optimum concentration for the liposome to take effect for inhibiting tumor cells (Additional file 1: Figure S1). Under TEM, the obtained ILA@Lip showed uniform sphere-like morphology (Fig. 1b and c). As measured by dynamic light scattering (DLS), the ILA@Lip showed uniform size distribution with a mean diameter of ∼90 nm (Fig. 1d), which was consistent with the TEM images. The encapsulation efficiencies and loading capacity of IR 780, Lenvatinib and AQ4N in ILA@Lip were quantified to be 59.79% ± 1.24, 45.11% ± 1.09 and 9.6% ± 0.34, respectively. The loading capacity of IR 780, lenvatinib and AQ4N in ILA@Lip were determined to be 1.85% ± 0.04, 1.37% ± 0.09 and 0.29% ± 0.03, respectively. Furthermore, by recording the characteristic absorbance of lenvatinib, AQ4N and IR780, it was determined that the release of lenvatinib, AQ4N and IR780 were slightly increased in acidic conditions or with laser irradiation (Additional file 1: Figure S2). Less than 20% of the agents were released from ILA@Lip in medium after incubation for 72 h. In order to evaluate the stability of ILA@Lip in tumor’s acidic environment, ILA@Lip was dissolved in PBS of different pH, and the size distribution was tested (Fig. 1e). In acidic PBS (pH = 2.5 or 5.5), the particle size of liposomes (~ 80 nm) is slightly smaller than in neutral PBS (pH = 7.4). This change may ascribe to the drug leakage in the lipid bilayer. The acidic environment induction of the drug release pattern revealed that ILA@Lip would be delivered in the lysosomes and taken effect inside tumor cells, which could raise the accuracy of treatment and reduce the potential toxicity. In addition, to investigate the store stability, ILA@Lip was dissolved in PBS and stored at 4 °C for two weeks. No significant change was detected in size distribution (Fig. 1f) and zeta potential (Fig. 1g) of the ILA@Lip. In the aim to compare the optical stability between free IR 780 and ILA@Lip, a UV–Vis-NIR spectrophotometer was used to measure the absorption of free IR 780 and ILA@Lip under laser (808 nm, 1.0 W cm^−2^) irradiation for 1 min (Additional file 1: Figure S3). Figure 1h showed the change in absorption intensity of the characteristic absorption peak of IR 780 and ILA@Lip after 808 nm laser irradiation for 1 min. Under laser radiation, the characteristic absorption peak intensity of free IR 780 decreased faster than ILA@Lip, indicating IR 780 encapsulated in liposomes exhibited improved photostability. ILA@Lip was dissolved in PBS, FBS, 1640, or DMEM, and stored at 4 °C for two weeks and the corresponding images were recorded without apparent change (Fig. 1i). These investigations confirmed that ILA@Lip had good stability in isotonic fluid with tissue fluid for long-term storage.

### The multifunctionality of ILA@Lip

**Fig. 2:**
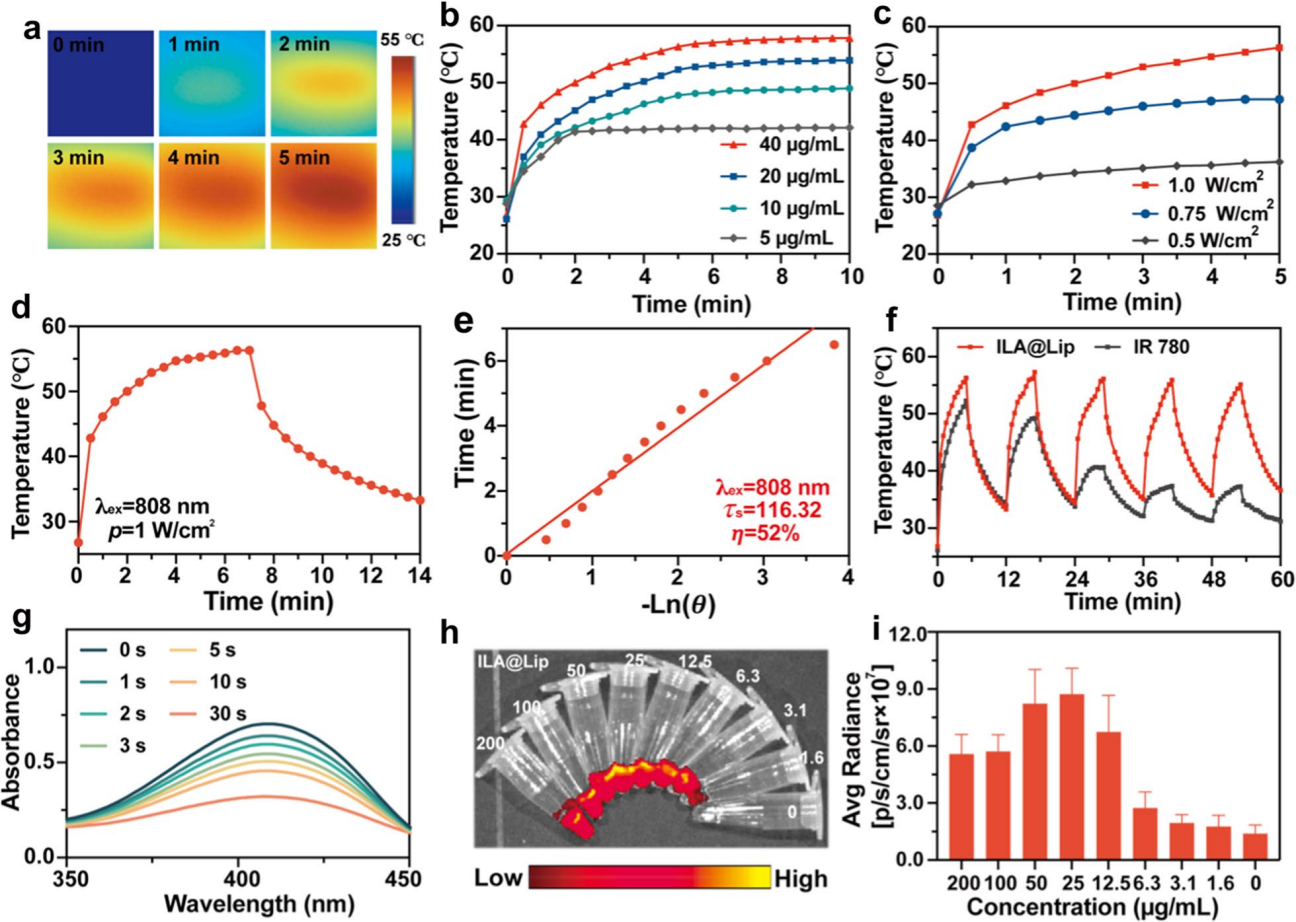
**a** IR thermal images of ILA@lip (20 μg mL^−1^) under 808 nm laser (1.0 W cm^−2^) irradiation at determined time points. **b** Temperature change profiles of ILA@Lip solution of different concentrations under laser (808 nm, 1.0 W cm^−2^) irradiation. **c** Temperature change curves of ILA@Lip solution (IR 780 = 20 μg mL^−1^) under 808 nm laser of different power irradiation. **d** Heating and cooling curves of ILA@Lip solution. **e** Linear time data obtained from the cooling period. **f** Temperature changes of IR 780 (IR 780 = 20 μg mL^−1^) and ILA@Lip (IR 780 = 20 μg mL^−1^) in five laser on/off cycles of laser irradiations (808 nm, 1.0 W cm^–2^). **g** UV–vis absorption spectrum of DPBF for detecting ROS generation ability of ILA@Lip under 808 nm laser (1.0 W cm^−2^) irradiation. **h** Fluorescence imaging of a series of concentrations of ILA@Lip solution in tubes. **i** Quantitative analysis of average radiance in ILA@Lip solution of different concentrations

As IR 780 is also an excellent photothermal agents, the photothermal performance of ILA@Lip was firstly examined by recording the temperature change using an infrared (IR) thermal camera during 808 nm laser irradiation periods (Fig. 2a). As expected, the ILA@Lip showed elevated temperature with increasing concentration (Fig. 2b), irradiation power density (Fig. 2c), and irradiation time. Notably, the photothermal conversion efficiency of ILA was calculated to be 52% (Fig. 2d, e). These results indicate that ILA@Lip is a prospective candidate for PTT in vivo [42]. Additionally, the photothermal performance of free IR 780 decreased dramatically after just one cycle and then dropped gradually after 5 cycles of irradiation (Fig. 2f), which demonstrated that free IR 780 degraded rapidly. The photothermal performance of ILA@Lip did not exhibit attenuation marginally in the temperature-rising effect during five successive cycles of laser on/off irradiation, indicating the enhanced photostability of IR 780 in ILA@Lip with the well-preserved photothermal response after five repeated NIR laser exposures, in contrast to the rapid decomposition of free IR 780. The singlet oxygen (^1^O_2_) production of ILA@Lip under laser irradiation was then tested by DPBF as a probe, which could irreversibly react with ^1^O_2_ to decrease the UV absorption intensity. Noteworthily, the DPBF absorption (λ = 450 nm) intensity rapidly degraded under laser irradiation over time, indicating the ^1^O_2_ was rapidly generated after 808 nm laser (1.0 W cm^−2^) irradiation (Fig. 2g). By utilizing the SOSG, we found that this an obvious increase in the PDT-triggered ^1^O_2_ generation ability of IR 780 and ILA@Lip after 808 nm laser (1.0 W cm^−2^) irradiation for a series of periods (Additional file 1: Figure S4). Thus, the ILA@LIP could serve as an excellent agent for PDT treatment. To explore the fluorescence imaging ability of ILA@Lip, different concentrations of free IR 780 and ILA@Lip solution were examined in the IVIS spectrum (Fig. 2h, i, Additional file 1: Figure S5). ILA@Lip kept a dose-dependent fluorescence signal of IR 780. These results suggested the great fluorescence imaging potential of ILA@Lip in vivo.

### In vitro cellular uptake

**Fig. 3:**
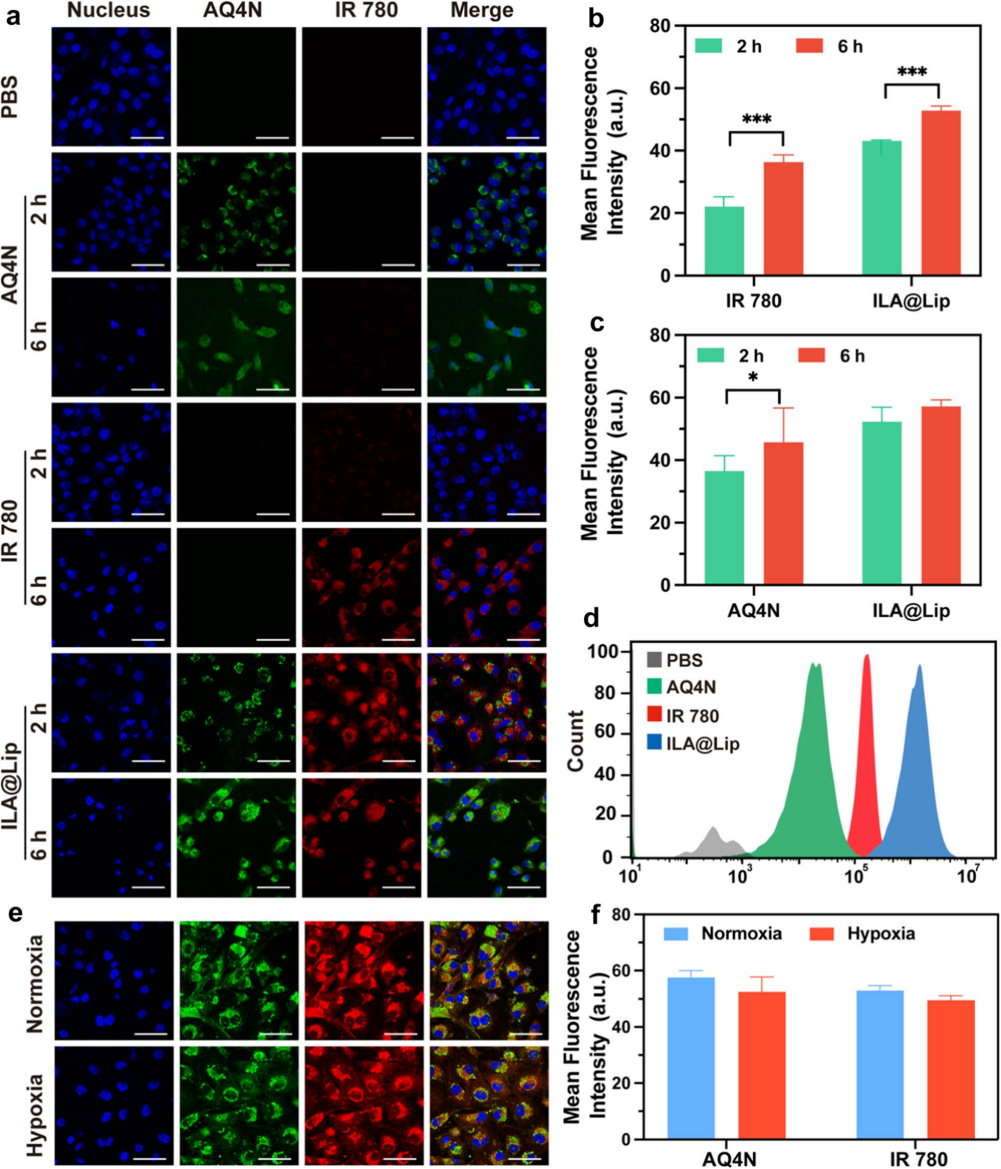
In vitro cell endocytosis of ILA@Lip. **a** Laser scanning confocal images of MDA-MB-231 cells treated with AQ4N (2 μg mL^−1^), free IR 780 (2 μg mL^−1^) and ILA@Lip (2 μg mL^−1^) for 2 and 6 h. DAPI: false-color blue signal, AQ4N: false-color green signal, IR 780: false-color red signal. Scale bars = 50 μm. Mean fluorescence intensity of **b** IR 780 and **c** AQ4N internalized by MDA-MB-231 cells after incubation for 2 and 6 h. Values represent means ± SD, n = 3. **P < 0.01; ***P < 0.001 vs control. **d** Flow cytometry analysis of MDA-MB-231 cells treated with AQ4N (2 μg mL^−1^), free IR 780 (2 μg mL^−1^), and ILA@Lip (2 μg mL^−1^) for 2 h. **e** Detailed distribution of MDA-MB-231 cells treated with ILA@Lip (2 μg mL^−1^) under normal conditions or hypoxic conditions for 4 h. Scale bars = 50 μm. **f** Mean fluorescence intensity of IR 780 and AQ4N internalized by MDA-MB-231 cells under normal conditions or hypoxic conditions for 4 h. Values represent means ± SD, n = 3

The cellular uptake behavior of ILA@Lip into MDA-MB-231 cells was carefully studied by recording the intrinsic fluorescence of IR 780 and AQ4N by using confocal microscopy (Fig. 3a). The cytosols of these MDA-MB-231 cells incubated with free IR 780 (Fig. 3b) and free AQ4N (Fig. 3c) exhibited time-dependent increment of fluorescence. Compared with the free IR 780 group, the intracellular IR 780 intensity was significantly higher in the ILA@Lip group even after incubation for only 2 h, because free IR 780 as a small lipophilic molecule could quickly diffuse through the cell membrane. While dropped in the liposome, the fluorescence of IR 780 appeared clearly in the cell after 2 h of incubation, which may be caused by the aggregation of liposomes in endosomes. The significantly stronger fluorescence of IR 780 in MDA-MB-231 cells indicated that ILA@Lip could increase the uptake of IR 780. At the same time, ILA@Lip accelerated AQ4N internalization speed as well. The quantitative results determined by flow cytometry analysis were in accordance with that of confocal results (Fig. 3d). The finding fully demonstrated that ILA@Lip could enhance cellular uptake and maintains this ability even in hypoxic conditions (Fig. 3e, f). Thereby the drug utilization was significantly improved and the dose requirement was reduced for in vivo application.

### Intracellular ROS analysis

**Fig. 4:**
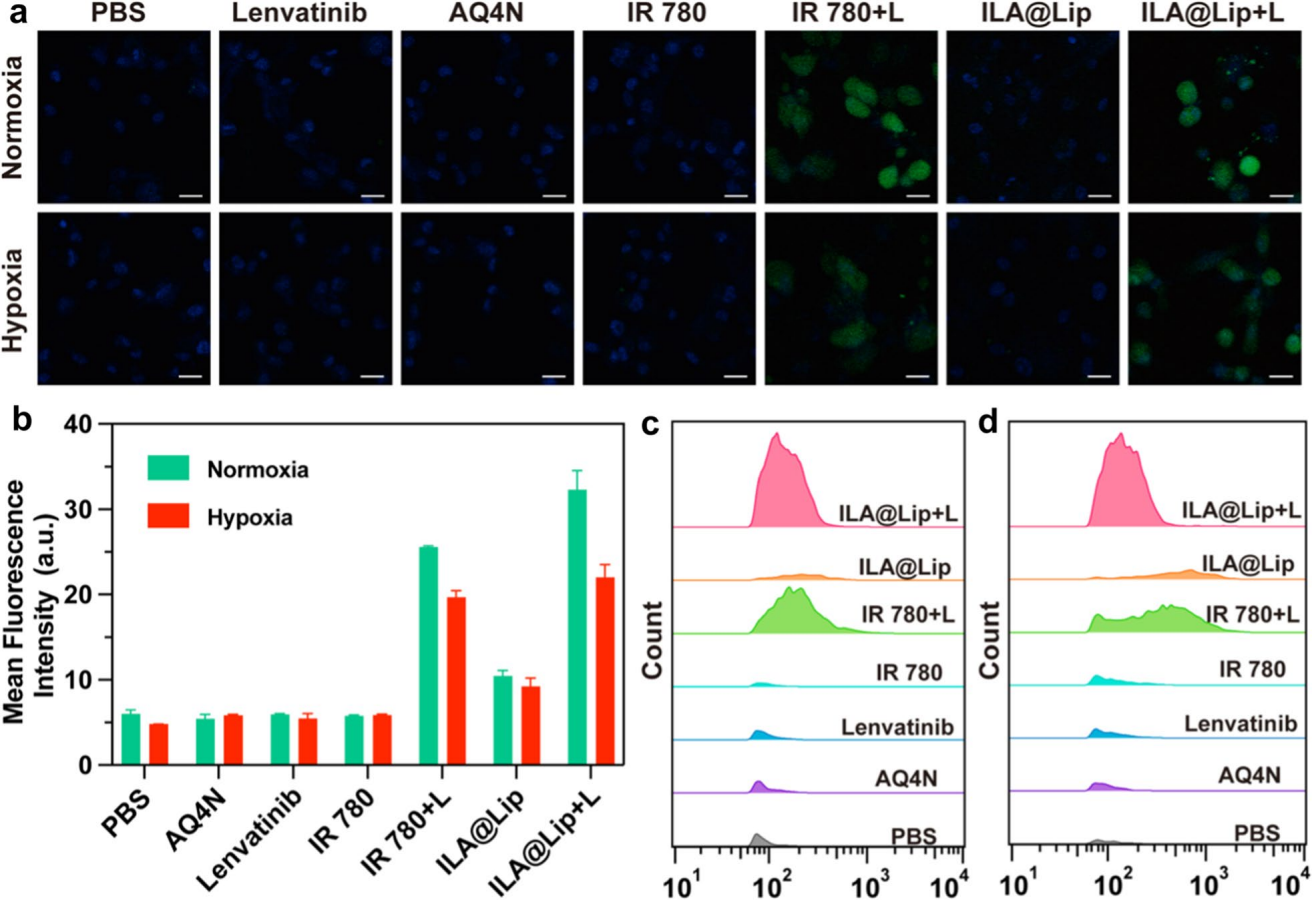
**a** Intracellular ROS generation of MDA-MB-231 cells treated with Lenvatinib (2 μg mL^−1^), AQ4N (2 μg mL^−1^), free IR 780 (2 μg mL^−1^) and ILA@Lip (2 μg mL^−1^) with or without laser (808 nm, 1.0 W cm^−2^, 3 min) irradiation was detected with DCFH-DA and observed by CLSM in normoxia or hypoxia. **b** Quantitative analysis of average mean fluorescence intensity ROS generation. Flow cytometric analysis of ROS production of MAD-MB-231 cells treated with PBS, Lenvatinib (2 μg mL^−1^), AQ4N (2 μg mL^−1^), free IR 780 (2 μg mL^−1^) and ILA@Lip (2 μg mL^−1^) with or without laser (808 nm, 1.0 W cm^−2^, 3 min) irradiation in **c** normoxia and **d** hypoxia

To evaluate ROS production of ILA@Lip in vitro, DCFH-DA probe was applied that can be degraded into DCFH in tumor cells, then can be oxidized by ROS to DCF, emitting green light [43]. Regardless of normoxic and hypoxic conditions, only DCFH-DA failed to generate ^1^O_2_ because of the lack of photosensitizer and light activation. The PDT efficiencies of lenvatinib, free IR 780, AQ4N, and ILA@Lip were compared by evaluating the generation levels of ROS after laser (808 nm, 1.0 W cm^−2^, 3 min) irradiation. As shown in Fig. 4a, no distinct green fluorescence was detected inside MDA-MB-231 cells after treatment with Lenvatinib, free IR 780, AQ4N. Brilliant green fluorescence was detected inside MDA-MB-231 cells after treatment with free IR 780 or ILA@Lip plus laser irradiation, validating ROS generation inside cancer cells. In addition, the level of ROS is much higher after incubation with ILA@Lip ascribed to the higher uptake efficiency of liposome, which led to more IR 780 accumulation inside the cells (Fig. 4a, b). However, due to photosensitizers needing molecular oxygen to transfer near-infrared (NIR) laser energy to form enough ROS, the transformation of molecular oxygen (O_2_) to transformation to ^1^O_2_ in hypoxia condition is limited [44]. Thus, we compared the intracellular ROS generation ability inside MDA-MB-231 cancer cells in hypoxic conditions and normoxia conditions. Both free IR 780 and ILA@Lip induced relatively lower ROS levels in hypoxic cells than in normoxia. However, compared to the no-laser groups or the no-photosensitizer group, IR 780 and ILA@Lip with laser irradiation produced enough ROS. In consideration of the transformation of environmental molecular O_2_ to ^1^O_2_ could induce hypoxia in the primary tumor, photodynamic function in effect will exacerbate hypoxia in the hypoxic tumor area and consume oxygen in the normoxic tumor. It has been reported that AQ4N can be reduced to the toxic form of AQ4 with remarkable increased affinity to nuclear DNA through a two-electron process mediated by the CYP3A member of the cytochrome P450 family under hypoxic conditions [45]. Therefore, ROS generation-induced hypoxia is beneficial for AQ4N activation, because either hypoxia areas in pre-existing tumors or newly generated hypoxia tumor areas will help AQ4N to convert to AQ4 and exert anti-tumor toxicity. In addition, the flow cytometry was further used to quantitatively detect ROS production in normoxia (Fig. 4c) and hypoxia (Fig. 4d), which were consistent with the CLSM result. The generation of ^1^O_2_ indicated that these ILA@Lip could also be served as a PDT agent for tumor elimination. And hypoxic environments with a limiting effect on ROS production can contribute to improving AQ4 toxicity. The production of ROS in a normoxic environment can also consume molecular oxygen for hypoxia activation of AQ4.

### Synergistic cytotoxicity and apoptosis-inducing activity

**Fig. 5:**
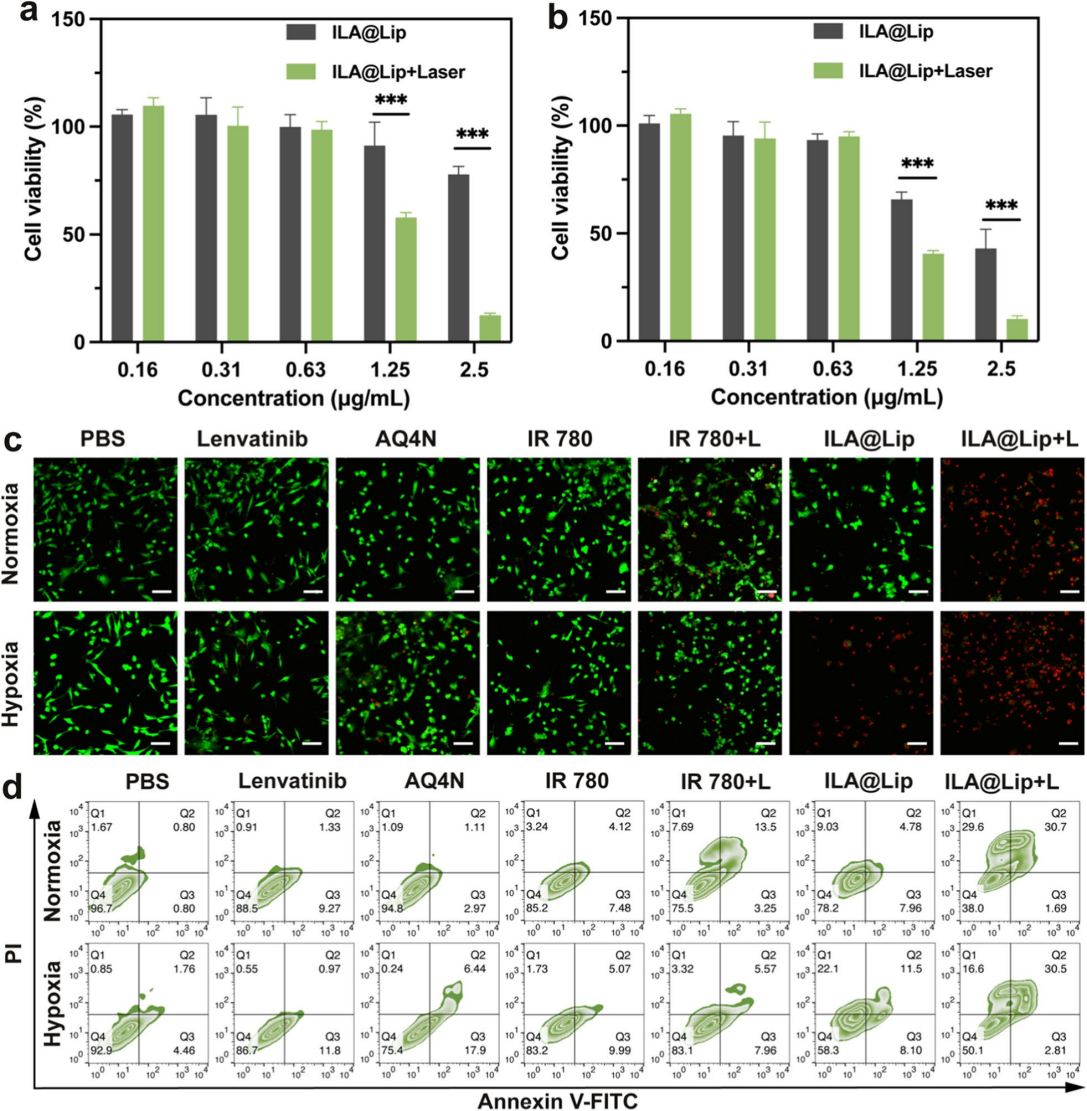
The viabilities of MDA-MB-231 cells after being treated with ILA@Lip and laser (808 nm, 1.0 W cm^−2^, 3 min) irradiation or not under **a** normoxia condition and **b** hypoxia condition were evaluated using CCK-8 assay. Values represent means ± SD, n = 3. ***p < 0.001. **c** Live/dead staining of MDA-MB-231 cells being treated with PBS, Lenvatinib (2 μg mL^−1^), AQ4N (2 μg mL^−1^), free IR 780 (2 μg mL^−1^) and ILA@Lip (2 μg mL^−1^) with or without laser irradiation in normoxia or hypoxia. Live cells and dead cells were signaled in green and red fluorescence, respectively. Scale bar = 100 μm. **d** The apoptosis detection of MDA-MB-231 cells treated by PBS, Lenvatinib (2 μg mL^−1^), AQ4N (2 μg mL^−1^), free IR 780 (2 μg mL^−1^) and ILA@Lip (2 μg mL^−1^) was analyzed by flow cytometry. *Q1* dead cells, *Q2* late apoptotic cells, *Q3* early apoptotic cells, *Q4* live cells

According to the above results, ILA@Lip presented excellent cellular uptake characteristics and remarkable PTT/PDT performances both in hypoxia and normoxia, and arouses the cytotoxicity of AQ4, thereby benefiting TNBC combination treatment. Subsequently, Both the hypoxia-activated cytotoxicity of AQ4N and the PTT/PDT cytotoxicity of IR 780 were evaluated with a CCK8 kit under normoxia or hypoxia. Firstly, the photodynamic cytotoxicity of ILA@Lip was evaluated with MDA-MB-231 cells. It was found that ILA@Lip (1.25 μg mL^−1^) with laser (808 nm, 1.0 W cm^−2^, 3 min) had PTT/PDT cytotoxicity to cells whether in normoxia or hypoxia environment (Fig. 5a, b). The cells only treated with ILA@Lip in hypoxia were killed as well, while the cells incubated with ILA@Lip in normoxia without laser irradiation were not obviously disturbed. Those results indicated that ILA@Lip has potent and selective cytotoxicity to cells in hypoxic environment, which can be ascribed to the hypoxia-activation of AQ4 toxicity. The toxicity of ILA@Lip to normal cells was determined by co-incubation of the ILA@Lip with HUVEC and L929 cells (Additional file 1: Figure S6). No significant toxicity was verified by CCK-8, which indicated good biocompatibility. The antitumor effect of ILA@Lip was further evaluated by living and dead cell staining through Calcein AM and PI (Fig. 5c and Additional file 1: Figure S7). Living cells with green fluorescence were flooded in the imaging field, while the MDA-MB-231 cells were treated with lenvatinib and IR 780 without irradiation regardless of normoxia or hypoxia after 8 h incubation with agents. MDA-MB-231 cells can be killed by AQ4 in hypoxia, but the killing effect was limited, and most MDA-MB-231 cells were killed after being treated with IR 780 with laser irradiation in normoxia, while half of the cells were still alive in hypoxia. This result was attributed to the limitation of PDT efficiency in hypoxia. In addition, almost cells were killed after being treated with ILA@Lip plus laser (808 nm, 1.0 W cm^−2^, 3 min) irradiation whether in hypoxic or normoxic conditions. The cytotoxicity ILA@Lip was maintained in hypoxia can be ascribed to the enhancement of the hypoxia-activated cytotoxicity of AQ4, which complemented the effect of reduced PDT efficiency in hypoxic conditions. Cell apoptosis was also quantitatively analyzed through Annexin V-FITC/PI assays (Fig. 5d) and the results were consistent with previous results. The percentage of killed and injured MDA-MB-231 cells in the ILA@Lip with laser irradiation group was far more than in other control groups. These results demonstrated that ILA@LIP showed effective photodynamic cytotoxicity and hypoxia-activated cytotoxicity, making it promising for PDT enhanced hypoxia-activated cancer therapy.

### In vitro angiogenesis and tube broken

**Fig. 6:**
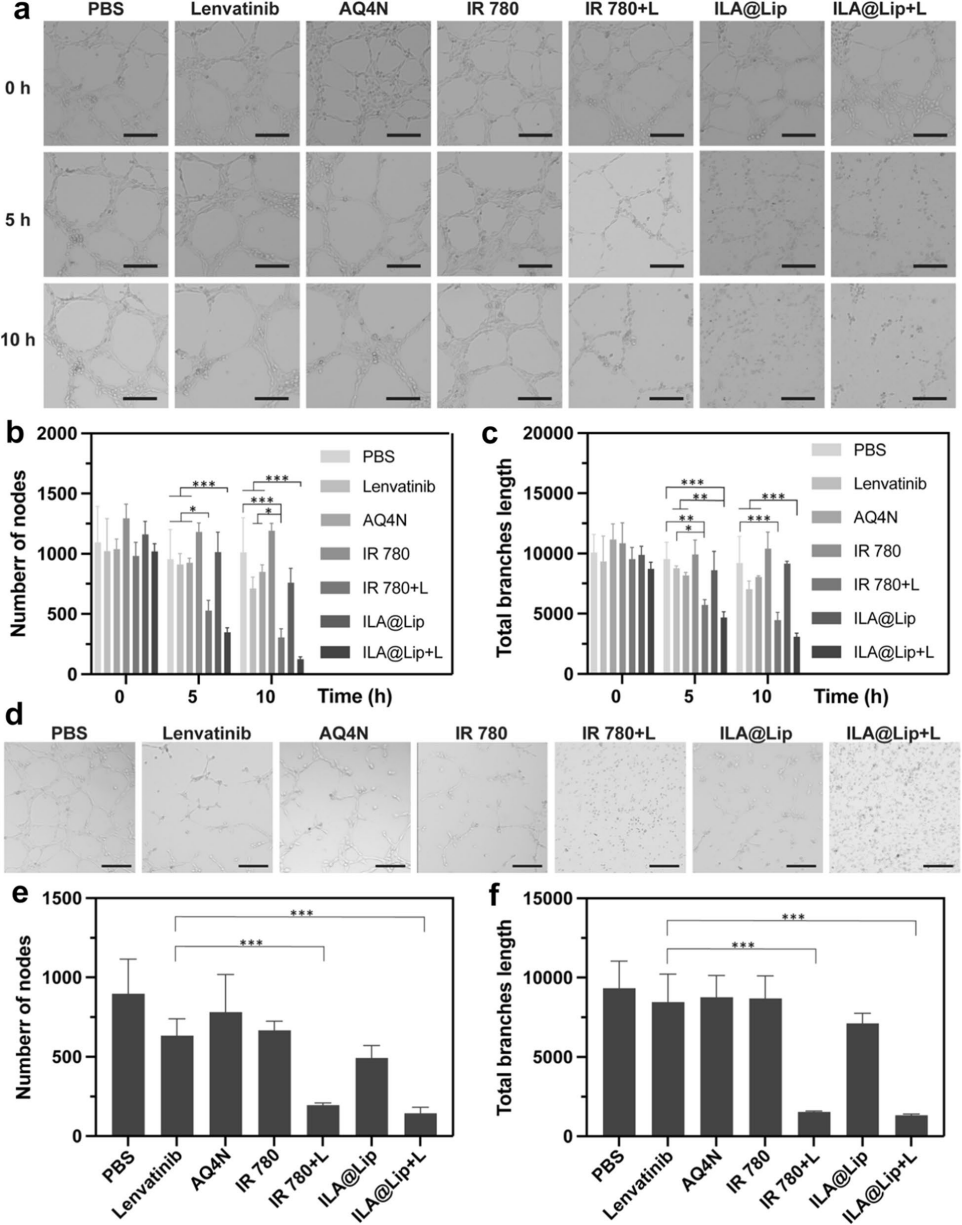
In vitro anti-angiogenesis assay. **a** Representative images of tube-like structures treated by Lenvatinib (2 μg mL^−1^), AQ4N (2 μg mL^−1^), free IR 780 (2 μg mL^−1^) and ILA@Lip (2 μg mL^−1^) with/without laser after 5 and 10 h. Scale bar = 200 μm. Quantitative analysis of **b** the average number of nodes and **c** total branch length after being treated by lenvatinib, AQ4N, IR 780, and ILA@Lip with/without laser after 5 and 10 h. Values represent means ± SD, n = 3. *P < 0.05; **P < 0.01; ***P < 0.001 vs control. **d** The microscope images show tube formation by HUVEC on Matrixgel after treatment with Lenvatinib (2 μg mL^−1^), AQ4N (2 μg mL^−1^), free IR 780 (2 μg mL^−1^) and ILA@Lip (2 μg mL^−1^) with/without laser for 5 h. Scale bar = 200 μm. Quantitative analysis of **e** the average number of nodes and **f** total branch length formatted after being treated by lenvatinib, AQ4N, IR 780, and ILA@Lip with/without laser after 5 h. Values represent means ± SD, n = 3. ***P < 0.001 vs control

To further assess the anti-angiogenic effect of ILA@Lip, HUVEC cell tube formation, and anti-angiogenesis assay were performed. As shown in Fig. 6a, b, c, after the cell tube formation was complete, AQ4N, Lenvatinib, free IR 780, and ILA@Lip were added into these cell tubes, and cells were irradiated with 808 laser (1.0 W cm^−2^, 3 min) in IR 780 and ILA@Lip plus laser group. As time went by, the blood vessel-like structure gradually decomposed. After 5 h, the vessel structure in IR 780 + laser, ILA@Lip, and ILA@ + laser groups began to break up. The blood vessels in the other control group presented the tendency to grow naturally. After 10 h, almost no vessel structure was observed in IR 780 and ILA@Lip plus laser group, indicating that photothermal effect can effectively destroy the cell tubes. By contrast, the tube only treated by ILA@Lip was broken, while the tube in IR 780 was nearly kept intact. These results indicated that ILA@Lip can effectively destroy the cell tubes via anti-angiogenesis, which can be ascribed to the presence of lenvatinib. After examining the ability of the various treatments to destroy the generated blood vessels, we also evaluated if the treatment could inhibit angiogenesis. For this, HUVECs were treated with AQ4N, lenvatinib, IR 780, and ILA@Lip with or without laser irradiation for 8 h in advance. Then the HUVEC cells were collected and cultured on Matrigel to monitor the tube formation (Fig. 6d, e, f). The node number and tube branch length in IR 780 and ILA@Lip with laser group were significantly less than the lenvatinib treated group, which is consistent with the results of the tube broken. No significant difference was detected between groups lenvatinib and ILA@Lip, indicating ILA@Lip maintained the anti-angiogenic effect of lenvatinib. In addition, Immunofluorescence was applied to study the mechanism of lenvatinib as an anti-angiogenic agent. Lenvatinib and ILA@lip anti-angiogenic by inhibiting the phosphorylation of VEGFR2 induced by VEGF in vascular endothelial cells (Additional file 1: Figure S8). In conclusion, ILA@Lip can be used for anti-angiogenic therapy, which benefited the hypoxia areas generation thereby the increased hypoxic area will contribute to AQ4 toxicity efficiency.

### In vivo imaging

**Fig. 7:**
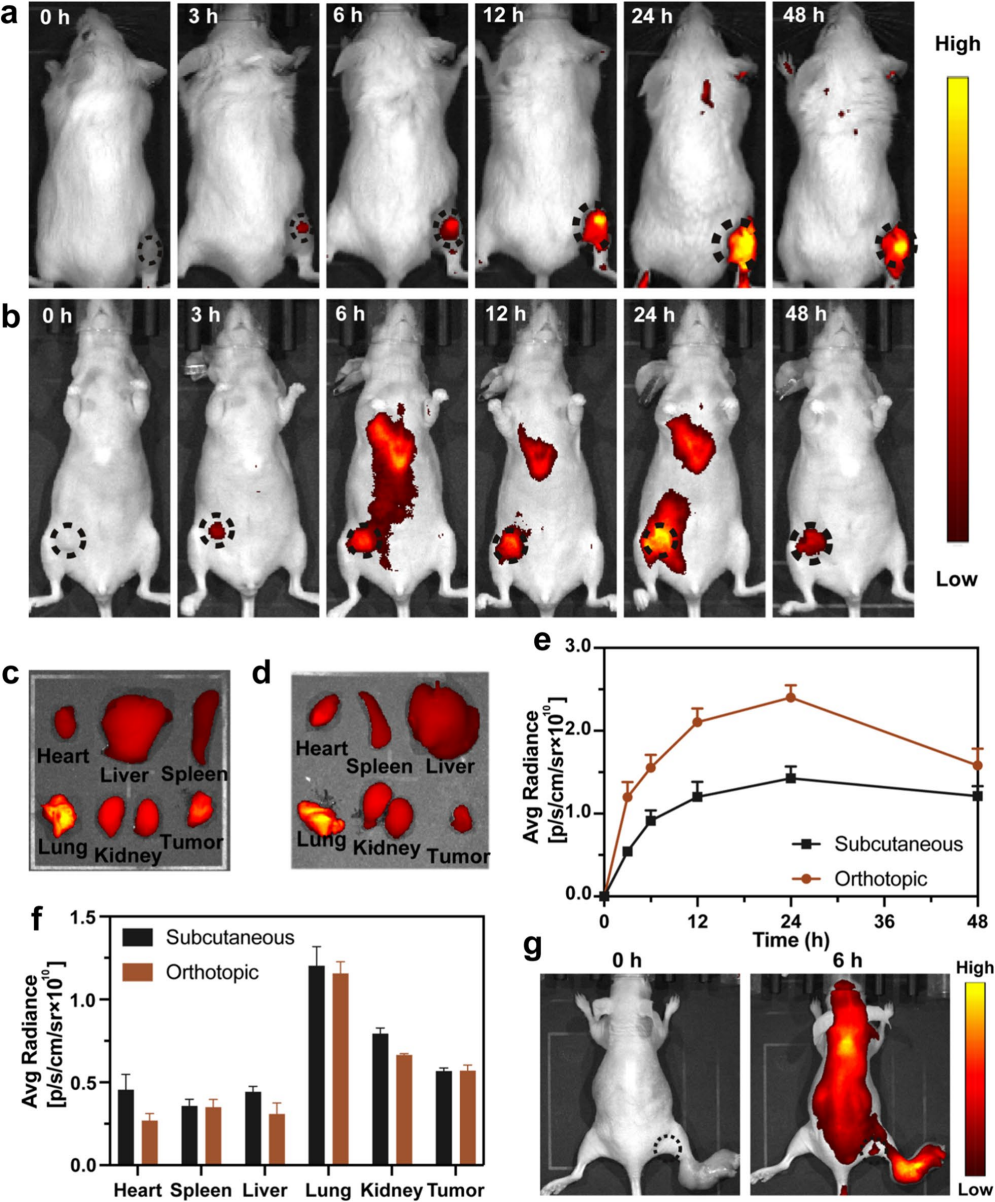
**a** In vivo real-time self-monitoring of drug distribution by observing fluorescence changes of ILA@Lip (AQ4N: 5 mg kg^−1^, Lenvatinib: 100 mg kg^−1^, IR 780: 5 mg kg^−1^) in subcutaneous 4T1 breast tumor-bearing mouse model. **b** In vivo real-time self-monitoring of drug distribution by observing fluorescence changes of ILA@Lip (AQ4N: 5 mg kg^−1^, Lenvatinib: 100 mg kg^−1^, IR 780: 5 mg kg^−1^) in orthotopic 4T1 breast tumor-bearing mouse model. **c** Fluorescence distribution of ILA@Lip in tumor and key organs from subcutaneous 4T1 breast tumor-bearing mouse model after 48 h post-injection. **d** Fluorescence distribution of ILA@Lip in tumor and key organs from and orthotopic 4T1 breast tumor-bearing mouse model after 48 h post-injection. **e** Statistical assay of the fluorescence intensity of the tumor region at 0, 3, 6, 12, 24, and 48 h after injection. Values represent means ± SD, n = 3. **f** Measurements of the fluorescence intensity of tumor and key organs. Values represent means ± SD, n = 3. **g** In vivo fluorescence images of 4T1 tumor metastasized to a lymph node. The images were acquired after tail vein injection of IAL@Lip for 6 h

The in vivo pharmacokinetics profiles of ILA@Lip were carefully studied by recording the fluorescence of IR 780. As observed under an IVIS® Lumina III in vivo fluorescence imaging system, it was found gradually increased IR 780 fluorescence in the subcutaneous 4T1 breast tumor-bearing mouse model (Fig. 7a and Additional file 1: Figure S9) and orthotopic 4T1 breast tumor-bearing mouse (Fig. 7b and Additional file 1: Figure S9) with intravenous injection of ILA@Lip via tail. By quantitatively analyzing the region of interest (ROI) of these fluorescence images, the tumor accumulation of ILA@Lip was determined to reach the maximum post-administration for 24 h (Fig. 7e), and kept at a high level without obvious dropping in the following 24 h of the monitoring process, indicating the efficient tumor homing capacity of ILA@Lip in both subcutaneous and orthotopic 4T1 breast tumor models. In order to confirm tumor accumulation efficiency, 24 h later, the organs and tumor were isolated for ex vivo fluorescence imaging (Fig. 7c, d, and f). ILA@Lip accumulated in tumor tissues and displayed higher signals than heart, liver, and spleen, which also provides evidence for follow-up treatment. According to the pharmacokinetics and biodistribution of ILA@Lip in the subcutaneous and orthotopic TNBC model, the mice were intravenously injected with the free IR 780 or ILA@Lip and exposed to an 808 nm laser (1 W cm^−2^, 10 min) at 24 h post-administration. Considering the possibility of lymph node metastasis in TNBC and in order to further study the tumor imaging properties of ILA@Lip, a tumor lymph node metastasis model was established to validate imaging-guided surgical excision. Figure 7g showed the tumor in the lymph node could be discerned after injecting ILA@Lip for 6 h. This result indicated that the ILA@Lip could accumulate at the tumor metastatic lymph nodes for further diagnostic imaging of lymph node metastasis.

**Fig. 8:**
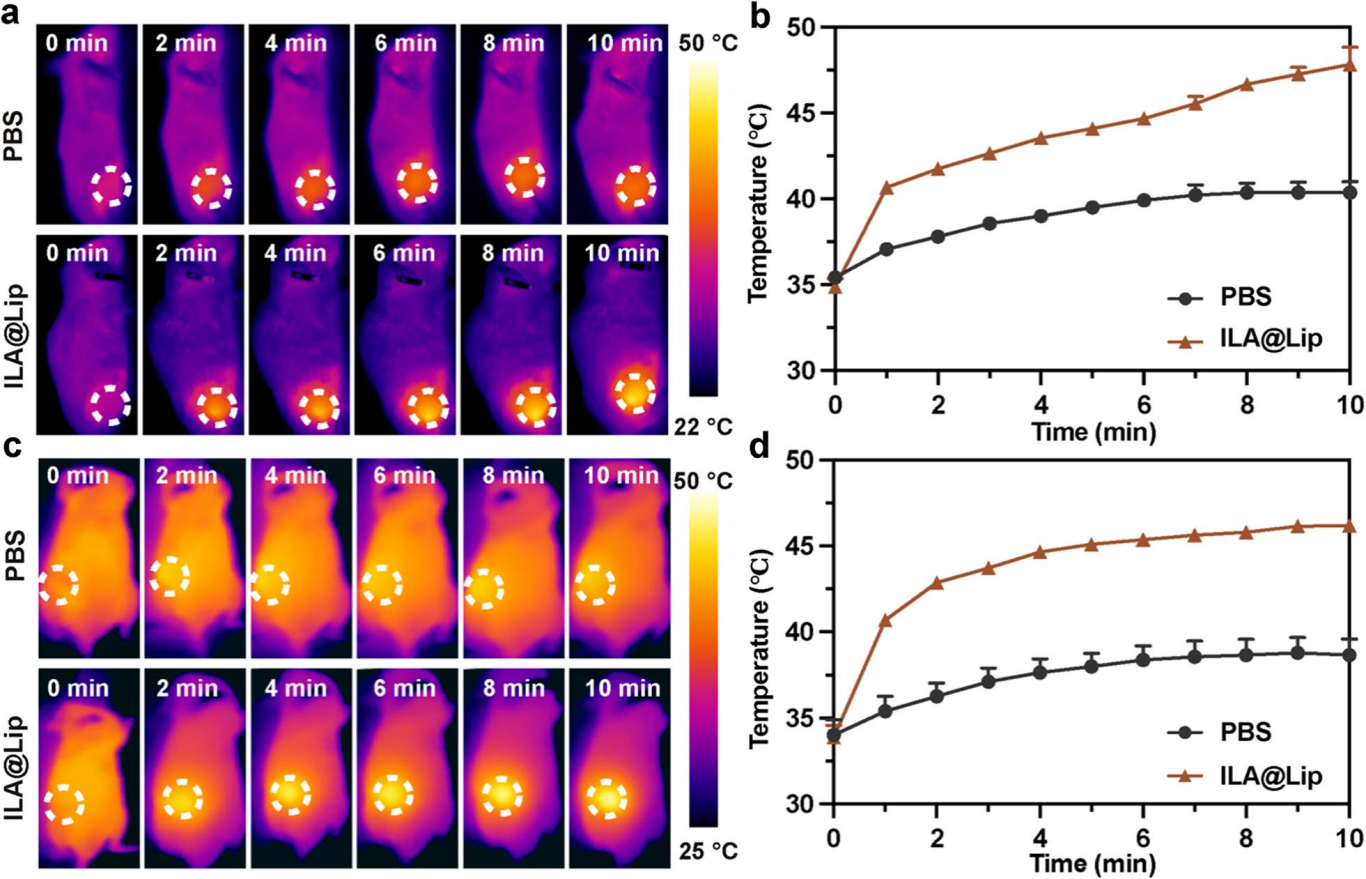
**a** IR thermal images of subcutaneous 4T1 tumor-bearing mice with intravenous injection of PBS and ILA@Lip (AQ4N: 5 mg kg^−1^, Lenvatinib: 100 mg kg^−1^, IR 780: 5 mg kg^−1^), **b** and their tumor temperature variations during a 10 min period of laser (808 nm, 1.0 W cm^−2^) irradiation at the tumor site. **c** IR thermal images of orthotopic 4T1 tumor-bearing nude mice with intravenous injection of PBS and ILA@Lip (AQ4N: 5 mg kg^−1^, Lenvatinib: 100 mg kg^−1^, IR 780: 5 mg kg^−1^), **d** and their tumor temperature variations during a 10 min period of laser (808 nm, 1.0 W cm^−2^) irradiation at the tumor site

When the drug reached its highest point (24 h post-administration) of accumulation in the tumor, we performed photothermal treatment on the tumor-bearing mice. The tumor temperature increase of the tumor site after injection of ILA@Lip in TNBC models was monitored during the laser irradiation period (Figs. 8a–d, Additional file 1: Figure S10). It can be seen that there is a significant PTT effect in the tumor area attributable to the outstanding tumor enrichment of ILA@Lip after 24 h of administration, which mean the good potential to destroy microvasculature. All of these results collectively demonstrated that ILA@Lip was a versatile probe for in vivo tracking and can efficiently accumulate in tumors via passive targeting to tumor, guiding to good photothermal treatment efficiency.

### In vivo therapeutic efficacy

**Fig. 9:**
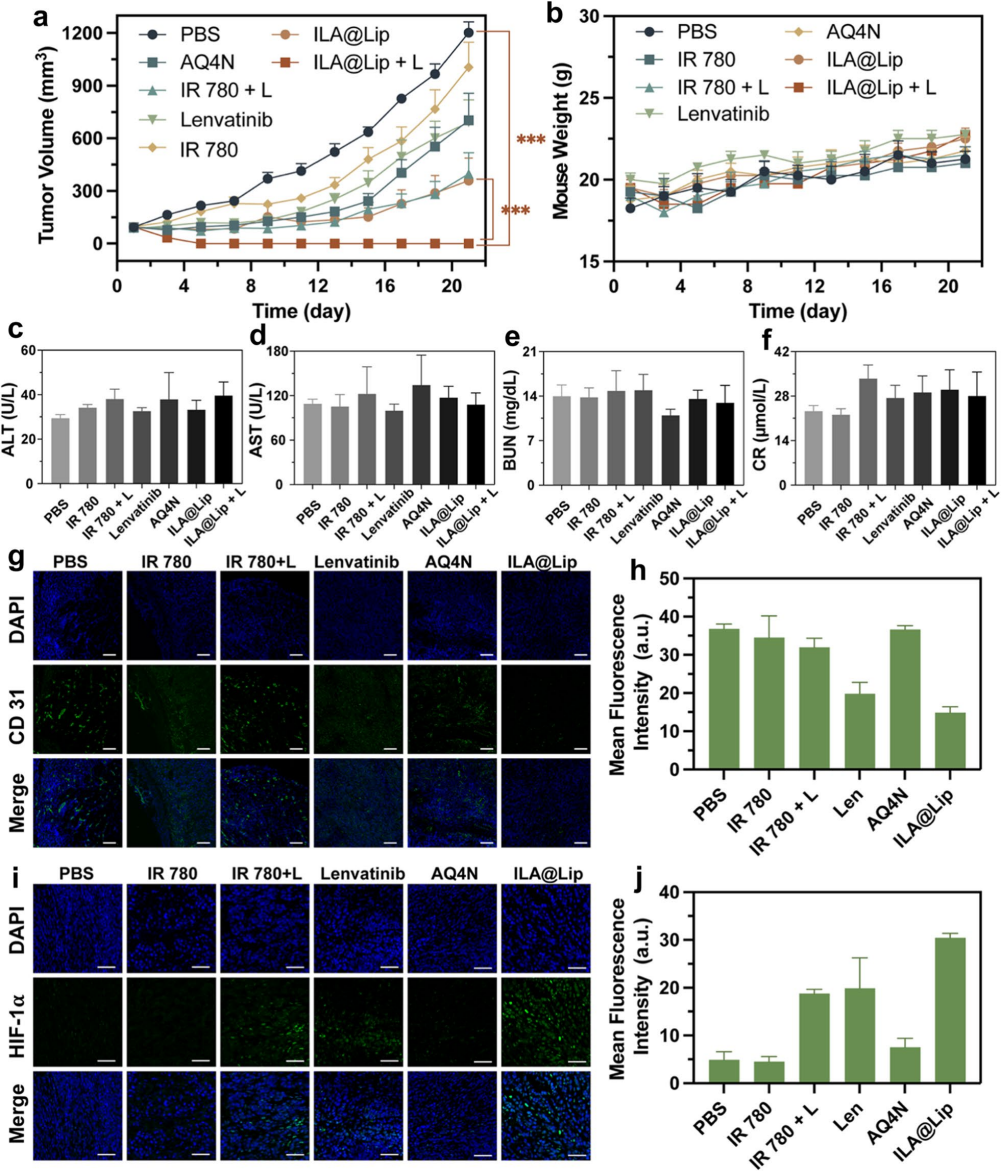
**a** The tumor growth curves of mice were intravenously treated with PBS, Lenvatinib (100 mg kg^−1^), AQ4N (5 mg kg^−1^), IR 780 (5 mg kg^−1^), and ILA@Lip (AQ4N: 5 mg kg^−1^, Lenvatinib: 100 mg kg^−1^, IR 780: 5 mg kg^−1^) with or without laser irradiation every 2 days for 3 cycles. Values represent means ± SD, n = 4. ***P < 0.001 vs control. **b** Body weight changes during the experiment. Values represent means ± SD, n = 4. Hepatic and renal function test of **c** ALT, d) AST, **e** BUN, and **f** CR. Values represent means ± SD, n = 4. **g** Immunofluorescence images and **h** Quantification for CD31 staining in 4T1 tumors of mice with different formulations at the experimental endpoint. Scale bar = 100 μm. Values represent means ± SD, n = 4. **i** Immunofluorescence images and **j** Quantification for HIF-1α staining in 4T1 tumors of mice with different formulations at the experimental endpoint. Scale bar = 50 μm. Values represent means ± SD, n = 4

With a good drug enrichment effect, the therapeutic potency of ILA@Lip with laser irradiation was evaluated on subcutaneous 4T1 tumor-bearing mouse models. A total of 28 female mice bearing subcutaneous 4T1 tumors were randomly divided into seven groups (n = 4) as below: (I) control group injected with PBS; (II) IR 780; (III) IR 780 + Laser; (IV) lenvatinib; (V) AQ4N; (VI) ILA@Lip; and (VII) ILA@Lip + Laser. The mice’s photothermal images and the tumors’ temperature-increasing curves had shown substantial PTT efficiencies of nanoparticles in vivo. By recording the tumor size using a digital caliper, we found that tumors in the mice treated with IR 780 + Laser, ILA@Lip, and ILA@Lip + laser were most effectively suppressed (Fig. 9a). In contrast to IR 780 + Laser, ILA@Lip, the ILA@Lip + laser treatments showed amazing inhibition effect on tumor growth, which led to complete elimination of tumor in the treatment endpoints, indicating the excellent anticancer effect achieved by good tumor accumulation, PTT efficiency enhanced by strengthened optical stability and improved cytotoxicity by hypoxia-activated AQ4. There was no noticeable body weight change among the groups (Fig. 9b). In addition, we carried out a pilot study to evaluate the safety profile of the ILA@Lip by analyzing critical indicators of renal and hepatic function, it was found that all treatments caused hardly change in AST, ALT, BUN, and CR (Fig. 9c–f). These results confirmed no obvious side effects of ILA@Lip. To examine the effect of ILA@Lip treatment on anti-angiogenesis and PDT effects-induced hypoxia, we stained tumor slices for CD31 (CD31 is a marker for vascular endothelial cells) and HIF-1α immunofluorescence staining. In vivo study, immunofluorescence staining results revealed that low concentrations of lenvatinib are not effective in anti-angiogenesis in vivo (Additional file 1: Figure S12). CD31-positive cells were significantly decreased after lenvatinib or ILA@Lip (Fig. 9g, h). We propose that lenvatinib was released from the liposomes under NIR irradiation and easily internalized by HUVEC. Thereby, lenvatinib can also exert a good anti-angiogenic effect in ILA@Lip + laser treated mice. In addition, the PTT of IR 780 + Laser would result in coagulative necrosis, thus destructing the tumor blood vessels and blocking the blood perfusion of tumors, which was in accordance with the lower CD31 signals in the tumor tissues. Furthermore, the effect of photodynamic treatment on tumor-bearing mice was further carefully evaluated by using endogenous hypoxia reporter of HIF-1α via immunofluorescence staining (Fig. 9i). Via semi-quantitative analysis (Fig. 9j), the positive hypoxia areas of this tumor were determined. The tumor slices of mice treated with PBS, IR 780 or AQ4N showed weak HIF-1α signal. The tumor slices of mice treated with IR 780 + laser, lenvatinib, and ILA@Lip displayed an increased level of HIF-1α expression, which was attributed to the oxygen depletion by ROS generation and inhibition of angiogenesis by Lenvatinib, respectively. Moreover, the increased expression of HIF-1α in ILA@Lip and Lenvatinib was negatively correlated with angiogenesis. Taken together, these results demonstrated that such mild photodynamic treatment and Lenvatinib could create an extremely hypoxic microenvironment, which was promising to benefit the hypoxia-activation of AQ4N. Overall, ILA@Lip with the properties of generation of ROS, the enhanced PTT, anti-angiogenesis induced hypoxia, and O_2_-consumption induced hypoxia for AQ4 activation was effective and safe. Besides, ILA@Lip can achieve multiple functions including in vivo imaging and potent anti-tumor activity.

The major organs and tumors were removed from sacrificed mice for further biochemical analysis as well. To further confirm the therapeutic effect, the proliferation levels and histological changes of tumors were analyzed in detail by utilizing both proliferation assay (Ki-67) and hematoxylin, respectively, in the tumor of the subcutaneous tumor-bearing mice (Additional file 1: Figures S13a, b). A significantly reduced positive rate was detected in the tumor from mice treated with IR 780 + laser and ILA@Lip. By contrast, the other four groups showed little or no change in proliferation positive rate in tumor slices. H and E stained slices of the major organs indicated no damage to normal tissues, while all slices exhibited normal membrane morphology and nuclear structures (Additional file 1: Figure S13c). However, the growth of metastatic 4T1 breast tumors in the liver was observed by H and E staining. According to H and E staining of the liver, many metastatic tumor nodules were observed in the livers of mice treated with PBS, lenvatinib, AQ4N, and IR 780 without laser irradiation. In contrast, no visible metastatic niches were found in the livers of mice treated with IR 780 + laser, and ILA@Lip regardless with or without laser irradiation. The H and E staining of liver resected from mice confirmed the ILA@Lip inhibition of cancer metastasis of 4T1. Compared to only one-way hypoxia-activated AQ4 (consumption of molecular O_2_ by PDT or anti-angiogenesis-induced limitation of O_2_ delivery), the synergy between the two ways hopefully plays a greater role in stimulating cytotoxicity of AQ4. These results suggested that the ILA@Lip + laser can elicit strong anticancer immunogenicity to remarkably promote survival rates through anti-angiogenesis and PDT-primed hypoxia-activated AQ4 toxicity.

### Antitumor effects in orthotopic TNBC

**Fig. 10:**
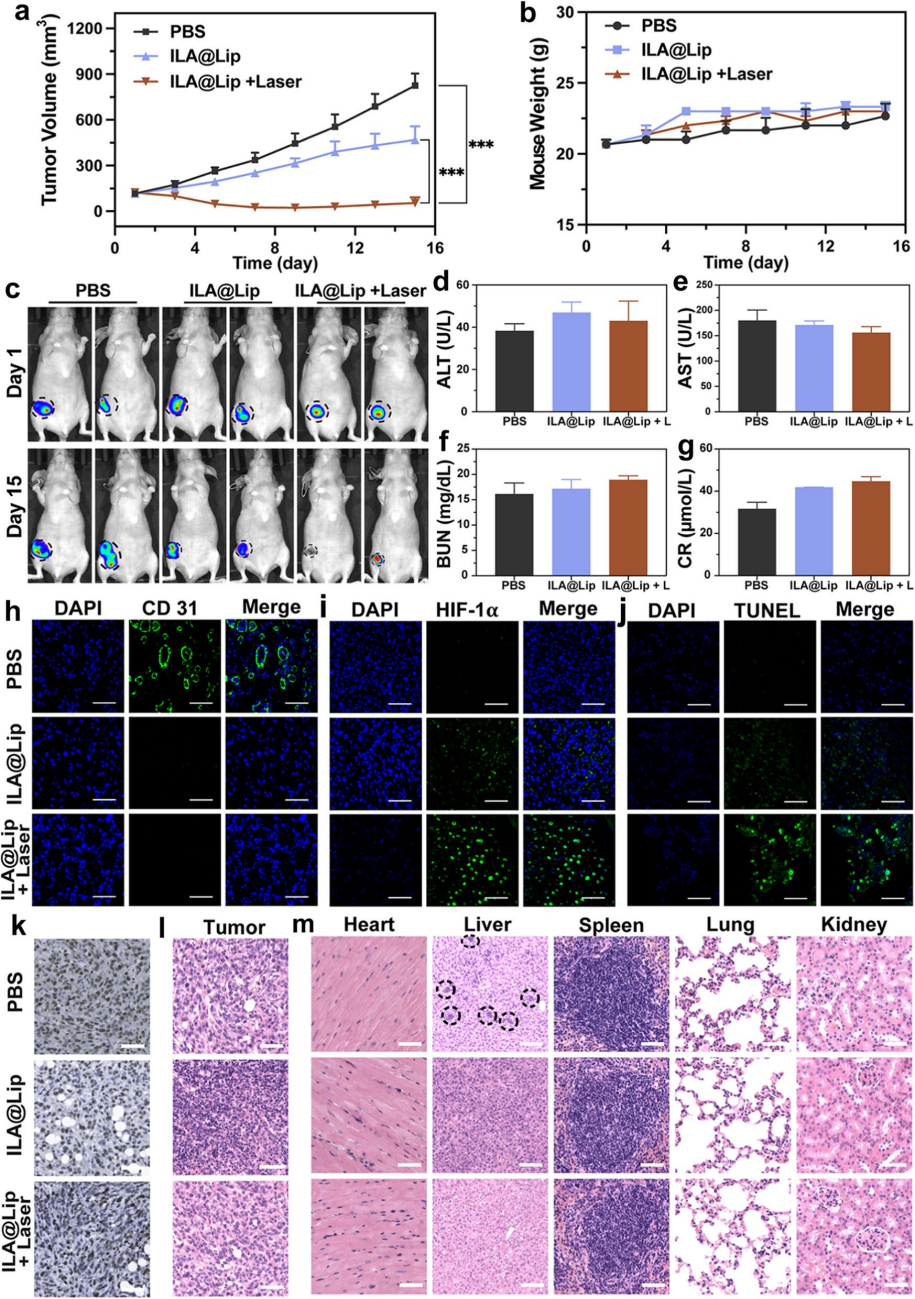
**a** The tumor growth curves of mice were intravenously treated with PBS, and ILA@Lip (AQ4N: 5 mg kg^−1^, Lenvatinib: 100 mg kg^−1^, IR 780: 5 mg kg^−1^) with or without laser irradiation every 2 days for 3 cycles. Values represent means ± SD, n = 3. ***P < 0.001 vs control. **b** Body weight changes during the experiment. Values represent means ± SD, n = 3. **c** Representative bioluminescence images of mice with tumors after treatments. Hepatic and renal function test of **d** ALT, **e** AST, **f** BUN, and **g** CR. Values represent means ± SD, n = 3. Representative immunofluorescence images of **h** CD 31 and **i** HIF-1α staining in 4T1 tumors of mice with different treatments at the experimental endpoint. Scale bar = 50 μm. **j** TUNEL staining of tumor slices from mice with different treatments. Scale bar = 50 μm. **k** The immunohistochemical studies of Ki-67. Scale bars = 50 μm. H&E staining of **l** tumor slices and **m** major organs from mice after various treatments. Circled areas are the tumor cells. Scale bars = 50 μm (tumor, heart, spleen, lung, kidney); Scale bars = 100 μm (liver)

In orthotopic 4T1 tumor-bearing mice were used to evaluate the in vivo therapeutic effects of ILA@Lip combined with laser irradiation treatment. The mice were inoculated with 4T1 tumors on the breast fat pad of female nude mice until the tumor volume was up to ~ 120 mm^3^. Similar to the therapy outcome in the subcutaneous 4T1 tumor-bearing mice, the tumor volume was significantly inhibited in ILA@Lip and ILA@Lip with laser irradiation groups (Fig. 10a and c, Additional file 1: Figure S11). The inhibition of tumors in the ILA@Lip group was attributed to the anti-angiogenic-induced hypoxia, which activated non-toxic AQ4N to toxic AQ4N. The tumor elimination was more pronounced in ILA@Lip with laser irradiation group, even one tumor in a mouse was eradicated. This excellent orthotopic TNBC tumor suppression effect resulted from the activation of AQ4N by tumor hypoxia due to the synergistic effect of PDT and anti-angiogenesis. Minor changes in the body weights of the treated mice were detected (Fig. 10b). No noticeable difference was caused with ILA@Lip with laser irradiation treatments in AST, ALT, BUN, and CR (Fig. 10d–g), which confirmed the excellent renal and hepatic safety profile of ILA@Lip in orthotopic TNBC.

To further visually identify the therapeutic efficacy, the expression levels of CD31 and HIF-1α in the tumor were analyzed. The vascular fractures in the ILA@Lip with or without laser irradiation groups were significantly lower than that in the PBS groups (Fig. 10h). The results suggested that the good accumulation of ILA@Lip in the tumor site holds high proficiency in the anti-angiogenesis effect. In addition, ILA@Lip laser treatment exhibited the best hypoxia induction ascribed to both the anti-angiogenesis and the oxygen consumption of PDT (Fig. 10i). The tumor slices were stained with the TUNEL to identify the apoptosis. The tumor cells in the ILA@Lip with laser irradiation stained strongly positive, indicating that the cell apoptosis was much stronger (Fig. 10j). In corresponding, tumors treated with ILA@Lip with laser irradiation showed negative Ki-67 staining, indicating that the proliferation of tumor cells was suppressed (Fig. 10k). All these data indicated the outstanding performance of hypoxia-activation chemotherapy led by the combination of anti-angiogenesis with PDT in orthotopic TNBC therapy. The main organs and tumors of mice were obtained and stained with H&E. As shown in Fig. 10l, m, tumor invasion to the livers in the PBS group could be witnessed. In contrast, no tumor invasion was observed in ILA@Lip treated mice. The above results suggested ILA@Lip can serve as a potential anti-tumor nano-strategy.

### Imaging-guided surgical removal of the tumor in lymph node

Identification and visualization of the TNBC lymph nodes metastasis are essential in precisely surgical resection of metastatic tumors. Tiny and early metastatic lymph nodes are not easily detected during traditional dissection [46]. Fluorescence-based imaging attracts attention due to its advantage of being non-invasively in living subjects, high sensitivity, and good biocompatibility [47]. Therefore, we used a model of metastatic lymph node tumor for elevating the fluorescence imaging-guided surgical resection of metastatic tumor. Based on the imaging results in Fig. 7g, the tumor at the lymph node can be surgically removed (Additional file 1: Figure S14a). Under NIR fluoroscopic imaging guidance, the epidermis at the lymph node was incised and the enlarged lymph node could be observed. The tumor was then surgically excised and the surface wound was sutured. The tumor bioluminescence signal at the lymph nodes disappeared after surgical excision (Additional file 1: Figure S14b). H and E staining of the obtained tumor sections also proved that the excised tissue was indeed a tumor metastasized to a lymph node (Additional file 1: Figure S14c). Therefore, guided by NIR imaging, the TNBC metastatic lymph node can show a complete incisal margin and be completely excised.

## Conclusion

In summary, the ILA@Lip theranostic nanosystem was successfully prepared by simultaneously encapsulating IR 780 as the photosensitizer and lenvatinib as an anti-angiogenic agent, together with an AQ4N molecule as the hypoxia-activated prodrug into the hydrophilic core. The ILA@Lip can be applied for the NIR fluorescence diagnostic imaging of TNBC and its lymph node metastasis for multimodal therapy. Lenvatinib in ILA@Lip inhibited angiogenesis and cut oxygen supply, thereby leading to enhanced hypoxia levels. Large amounts of ROS were produced while the IR 780 was irradiated by an 808 nm laser, which also rapidly exhausts oxygen in tumor cells to worsen tumor hypoxia. Through creating an extremely hypoxic in TNBC models, the conversion of non-toxic AQ4N to toxic AQ4 was much more efficient for hypoxia-activated chemotherapy. Cytotoxicity assay of ILA@Lip showed high toxicity in the hypoxic breast cancer cell, and the tumors at Balb/c mice treated by the ILA@Lip with laser irradiation were admirably suppressed in both subcutaneous tumor model and orthotopic tumor models. By utilizing ILA@Lip, we highlighted a profound strategy to create an extremely hypoxic TME to collectively benefit the therapeutic efficacy of hypoxia-activated chemotherapy, which realized collective suppression of tumor growth and has promising potential for the clinical TNBC treatment.

### Supplementary Information

**Additional file 1**: **Figure S1**. The viabilities of MDA-MB-231 cells after being treated with ILA@Lip with different loading ratios of agents under hypoxia condition. a) Under laser (808 nm, 1.0 W cm-2, 3 min) irradiation. b) No laser irradiation. Values represent means ± SD, n = 3. **Figure S2**. Time-dependent a) AQ4N, b) Lenvatinib, and c) IR 780 release profiles of ILA@Lip incubated in PBS solution with different pH values (pH 7.4, and 5.5) before and after laser irradiation. **Figure S3**. UV-vis spectrums of a) free IR 780, b) ILA@Lip after a series of time laser irradiation (808 nm, 1.0 W cm-2). **Figure S4**. Singlet oxygen generation abilities of IR-780 and ILA@Lip after different times of irradiation (808 nm, 1 W/cm2) were determined by using SOSG, whose recovered fluorescence indicated the generation of single oxygen. **Figure S5**. a) Fluorescence imaging of a series of concentrations of free IR 780 solution in the tube. b) Quantitative analysis of average radiance in free IR 780 solutions of different concentrations. **Figure S6**. The viabilities of a) HUVEC cells and b) L929 after being treated with ILA@Lip were evaluated using CCK-8 assay. Values represent means ± SD, n= 3. **Figure S7**. Quantitative analysis of Live/dead staining of MDA-MB-231 cells being treated with lenvatinib, AQ4N, IR 780, and ILA@Lip with or without laser irradiation in a) normoxia or b) hypoxia. Live cells and dead cells were signaled in green and red, respectively. (Mean ± S.D., n = 3). **Figure S8**. Lenvatinib inhibits the phosphorylation of VEGFR2 (p-VEGFR2) induced by VEGF in HUVEC. a) Immunofluorescence analysis of HUVEC cells labelling p-VEGFR2. Scale bar = 25 μm. b) Quantitative analysis of the p-VEGFR2 protein relative expression. (Mean ± S.D., n = 3). **Figure S9**. a) In vivo real-time self-monitoring of drug distribution by observing fluorescence changes of IR 780 (5 mg kg-1) in subcutaneous 4T1 breast tumor-bearing mouse model. b) In vivo real-time self-monitoring of drug distribution by observing fluorescence changes of IR 780 (5 mg kg-1) in orthotopic 4T1 breast tumor-bearing mouse model. **Figure S10**. a) IR thermal images of subcutaneous 4T1 tumor-bearing mice with intravenous injection of IR 780, b) and their tumor temperature variations during a 10 min period of laser (808 nm, 1.0 W cm-2) irradiation at the tumor site. **Figure S11**. Photos of tumors from orthotopic 4T1 breast tumor-bearing mice after being treated with PBS, and ILA@Lip with or without laser irradiation (808 nm, 1.0 W cm-2). **Figure S12**. Immunofluorescence images of CD31 staining in 4T1 tumors of mice with low concentration of lenvatinib. Scale bar = 50 μm. **Figure S13**. The immunohistochemical studies of a) Ki-67 and b) H and E of tumor cancer slices. The brown staining of the nucleus indicated the positive expression of Ki-67, and the higher the positive rate, the stronger proliferation of tumor cells. Scale bars = 50 μm. c) H and E staining of heart, liver, spleen, lung, and kidney from mice after various treatments. Circled areas are the tumor cells. Scale bars =50 μm. **Figure S14**. a) Photographs of a surgical procedure to remove a TNBC metastatic lymph node. b) Bioluminescence images of mice with lymph node metastasized tumors before and after surgical removal guided by NIR fluorescence images. c) H and E staining of tumor slices from surgically removed TNBC metastatic lymph node.

## Data Availability

All data analyzed during this study are included in this article and its supplementary information files.

## Abbreviation

TNBC: Triple-negative breast cancer
AQ4N: Banoxantrone
IR: Infrared
NIR: Near-infrared
ROS: Reactive oxygen species
ER: Estrogen receptor
PR: Progesterone receptor
HAPs: Hypoxia-activated prodrugs
AAT: Anti-angiogenic therapy
PDT: Photodynamic therapy
PTT: Photothermal therapy
ILA@Lip: IR780-Lenvatinib-AQ4N Lipsome
DOPE: 1,2-Dioleoyl-sn-glycero-3-phosphoethanolamine
DSPE-mPEG_2000_: 1,2-Distearoyl-sn-glycero-3-phosphoethanolamine-N-[methoxy (polyethylene glycol)-2000]
PBS: Phosphate buffered saline
TEM: Transmission electron microscopy
DLS: Dynamic light scattering
O_2_: Oxygen
^1^O_2_: Singlet oxygen
SOSG: Singlet oxygen sensor green
DCFH-DA: Dichloro-dihydro-fluorescein diacetate
DPBF: 1,3-Diphenylisobenzofuran
PI: Propidium iodide
ROI: Region of interest
AST: Aspartate aminotransferase
ALT: Alanine aminotransferase
BUN: Blood urea nitrogen
CR: Creatinine
HIF-1α: Hypoxia-induced factor alpha
H&E: Hematoxylin and eosin
DMEM: Dulbecco’s Modified Eagle medium
FBS: Fetal bovine serum
CCK-8: Cell counting kit-8
Ki 67: Histology and proliferation assay
