# Supplementary material for: NIR diagnostic imaging of triple-negative breast cancer and its lymph node metastasis for high-efficiency hypoxia-activated multimodal therapy
Source: J Nanobiotechnology. 2023 Sep 2;21:312. doi: 10.1186/s12951-023-02010-1 (PMC10475188; doi:10.1186/s12951-023-02010-1)
Supplement: Supplementary file 1 — Additional file 1: Figure S1. The viabilities of MDA-MB-231 cells after being treated with ILA@Lip with different loading ratios of agents under hypoxia condition. a) Under laser (808 nm, 1.0 W cm-2, 3 min) irradiation. b) No laser irradiation. Values represent means ± SD, n = 3. Figure S2. Time-dependent a) AQ4N, b) Lenvatinib, and c) IR 780 release profiles of ILA@Lip incubated in PBS solution with different pH values (pH 7.4, and 5.5) before and after laser irradiation. Figure S3. UV-vis spectrums of a) free IR 780, b) ILA@Lip after a series of time laser irradiation (808 nm, 1.0 W cm-2). Figure S4. Singlet oxygen generation abilities of IR-780 and ILA@Lip after different times of irradiation (808 nm, 1 W/cm2) were determined by using SOSG, whose recovered fluorescence indicated the generation of single oxygen. Figure S5. a) Fluorescence imaging of a series of concentrations of free IR 780 solution in the tube. b) Quantitative analysis of average radiance in free IR 780 solutions of different concentrations. Figure S6. The viabilities of a) HUVEC cells and b) L929 after being treated with ILA@Lip were evaluated using CCK-8 assay. Values represent means ± SD, n= 3. Figure S7. Quantitative analysis of Live/dead staining of MDA-MB-231 cells being treated with lenvatinib, AQ4N, IR 780, and ILA@Lip with or without laser irradiation in a) normoxia or b) hypoxia. Live cells and dead cells were signaled in green and red, respectively. (Mean ± S.D., n = 3). Figure S8. Lenvatinib inhibits the phosphorylation of VEGFR2 (p-VEGFR2) induced by VEGF in HUVEC. a) Immunofluorescence analysis of HUVEC cells labelling p-VEGFR2. Scale bar = 25 μm. b) Quantitative analysis of the p-VEGFR2 protein relative expression. (Mean ± S.D., n = 3). Figure S9. a) In vivo real-time self-monitoring of drug distribution by observing fluorescence changes of IR 780 (5 mg kg-1) in subcutaneous 4T1 breast tumor-bearing mouse model. b) In vivo real-time self-monitoring of drug distribution by observing [file 12951_2023_2010_MOESM1_ESM.docx]

Additional file

**NIR diagnostic imaging of triple-negative breast cancer and its lymph node metastasis for high-efficiency hypoxia-activated multimodal therapy**

*Yi Pan^1,2,3^, Longcai Liu^1,3,4^, Yichen He^1,2,3^, Luyi Ye^1,3,4^, Xin Zhao^1,3,4^, Zhiming Hu*^5^, Xiaozhou Mou*^1,3^, Yu Cai*^1,3^*

*^1^ Center for Rehabilitation Medicine, Rehabilitation & Sports Medicine Research Institute of Zhejiang Province, Department of Rehabilitation Medicine, Cancer Center, Zhejiang Provincial People's Hospital, Affiliated People's Hospital, Hangzhou Medical College, Hangzhou, Zhejiang, China, 310014.*

*^2^ College of Pharmacy, Zhejiang University of Technology, Hangzhou, Zhejiang, China, 310014.*

*^3^ Clinical Research Institute, Zhejiang Provincial People’s Hospital, Affiliated People’s Hospital, Hangzhou Medical College, Hangzhou, Zhejiang, China, 310014.*

*^4^ College of Pharmacy, Hangzhou Medical College, Hangzhou, China, 310059.*

*^5^ Department of Hepatobiliary Pancreatic Surgery, Zhejiang Provincial Tongde Hospital, Hangzhou, Zhejiang, China, 310012.*

E-mail: [mouxz@zju.edu.cn](mailto:mouxz@zju.edu.cn), [huzhiming4199@163.com](mailto:huzhiming4199@163.com), [caiyu@hmc.edu.cn](mailto:caiyu@hmc.edu.cn)


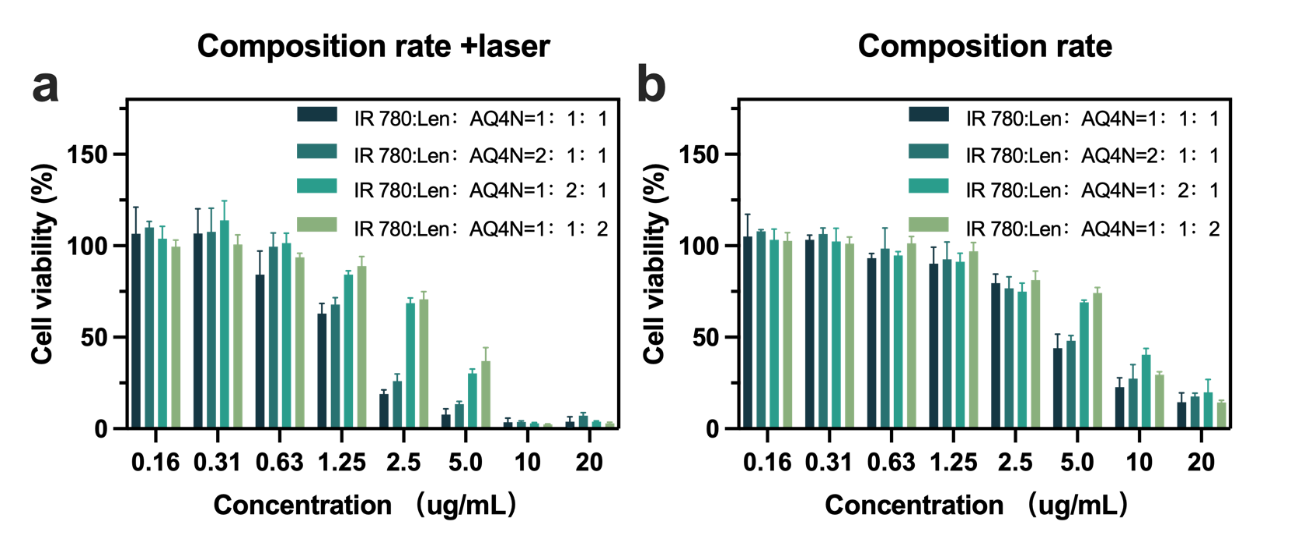


**Figure S1.** The viabilities of MDA-MB-231 cells after being treated with ILA@Lip with different loading ratios of agents under hypoxia condition. a) Under laser (808 nm, 1.0 W cm^-2^, 3 min) irradiation. b) No laser irradiation. Values represent means ± SD, n = 3.


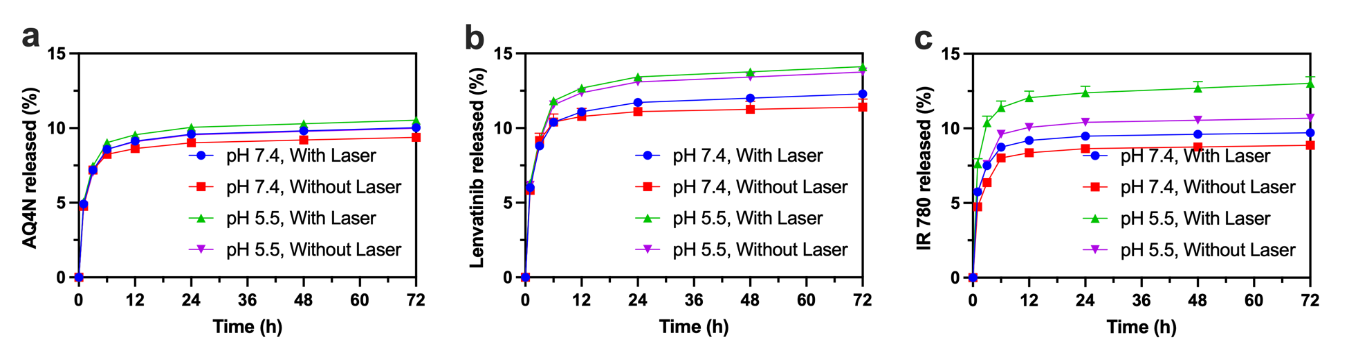


**Figure S2.** Time-dependent a) AQ4N, b) Lenvatinib, and c) IR 780 release profiles of ILA@Lip incubated in PBS solution with different pH values (pH 7.4, and 5.5) before and after laser irradiation.


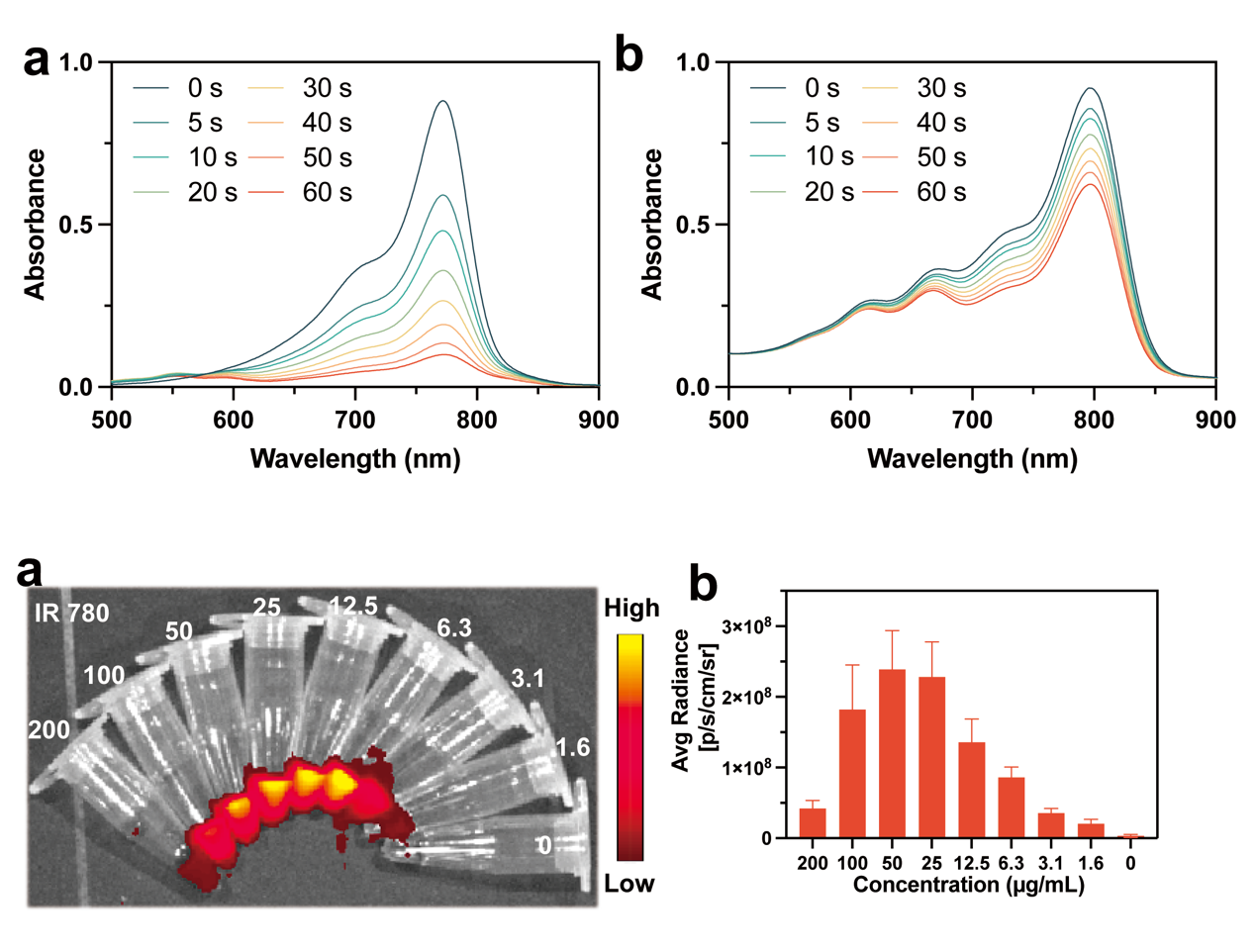


**Figure S3.** UV-vis spectrums of a) free IR 780, b) ILA@Lip after a series of time laser irradiation (808 nm, 1.0 W cm^-2^).


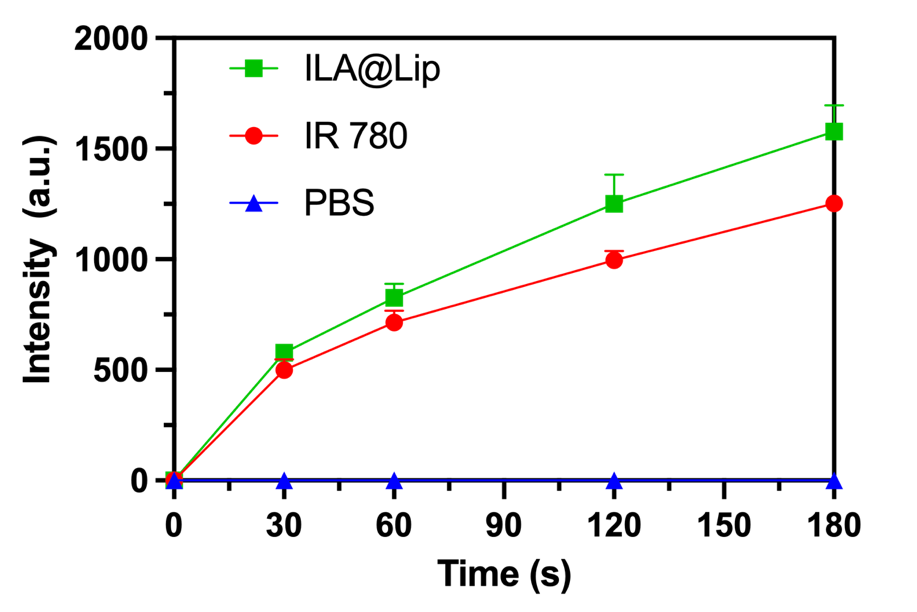


**Figure S4.** Singlet oxygen generation abilities of IR-780 and ILA@Lip after different times of irradiation (808 nm, 1 W/cm^2^) were determined by using SOSG, whose recovered ﬂuorescence indicated the generation of single oxygen.


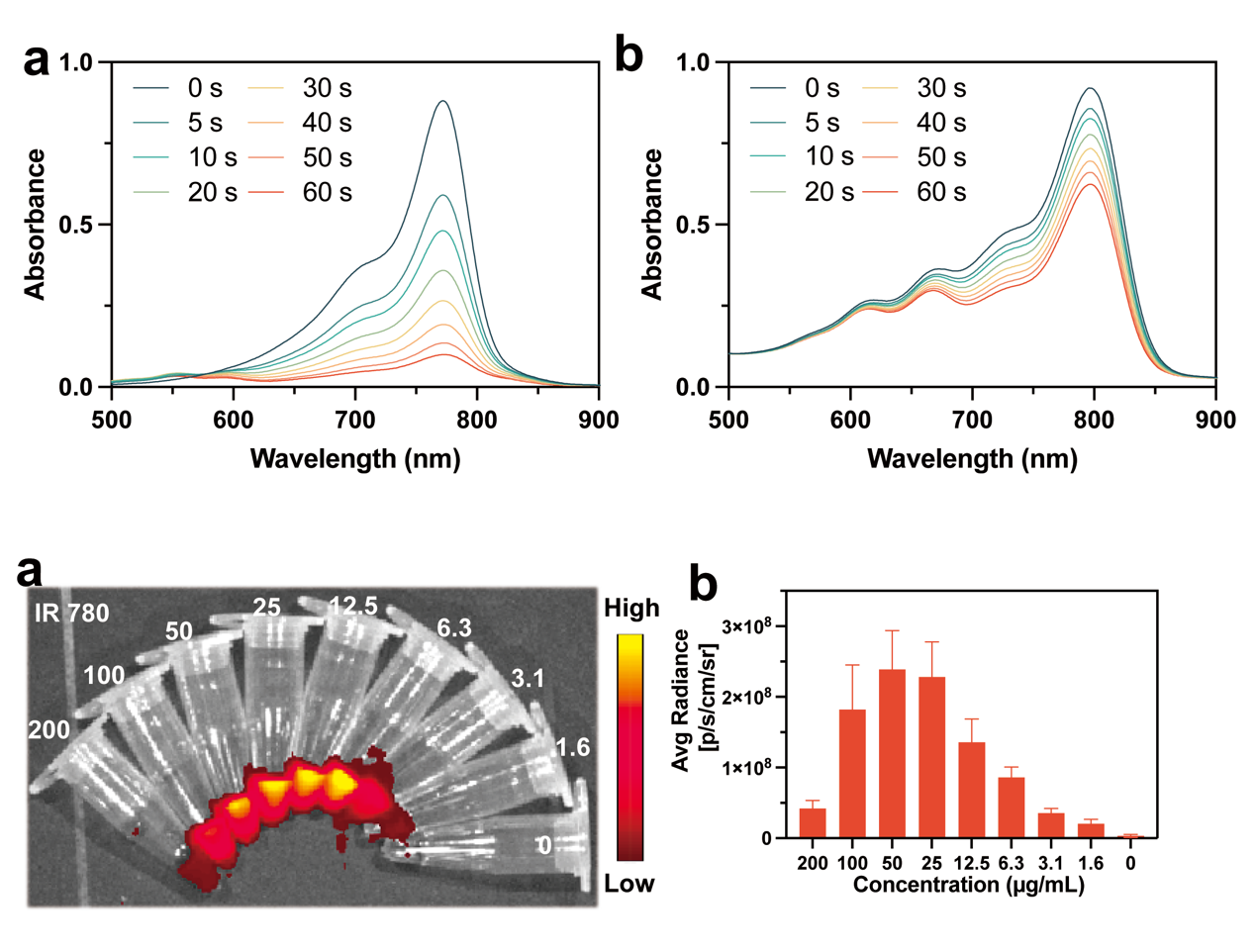


**Figure S5.** a) Fluorescence imaging of a series of concentrations of free IR 780 solution in the tube. b) Quantitative analysis of average radiance in free IR 780 solutions of different concentrations.


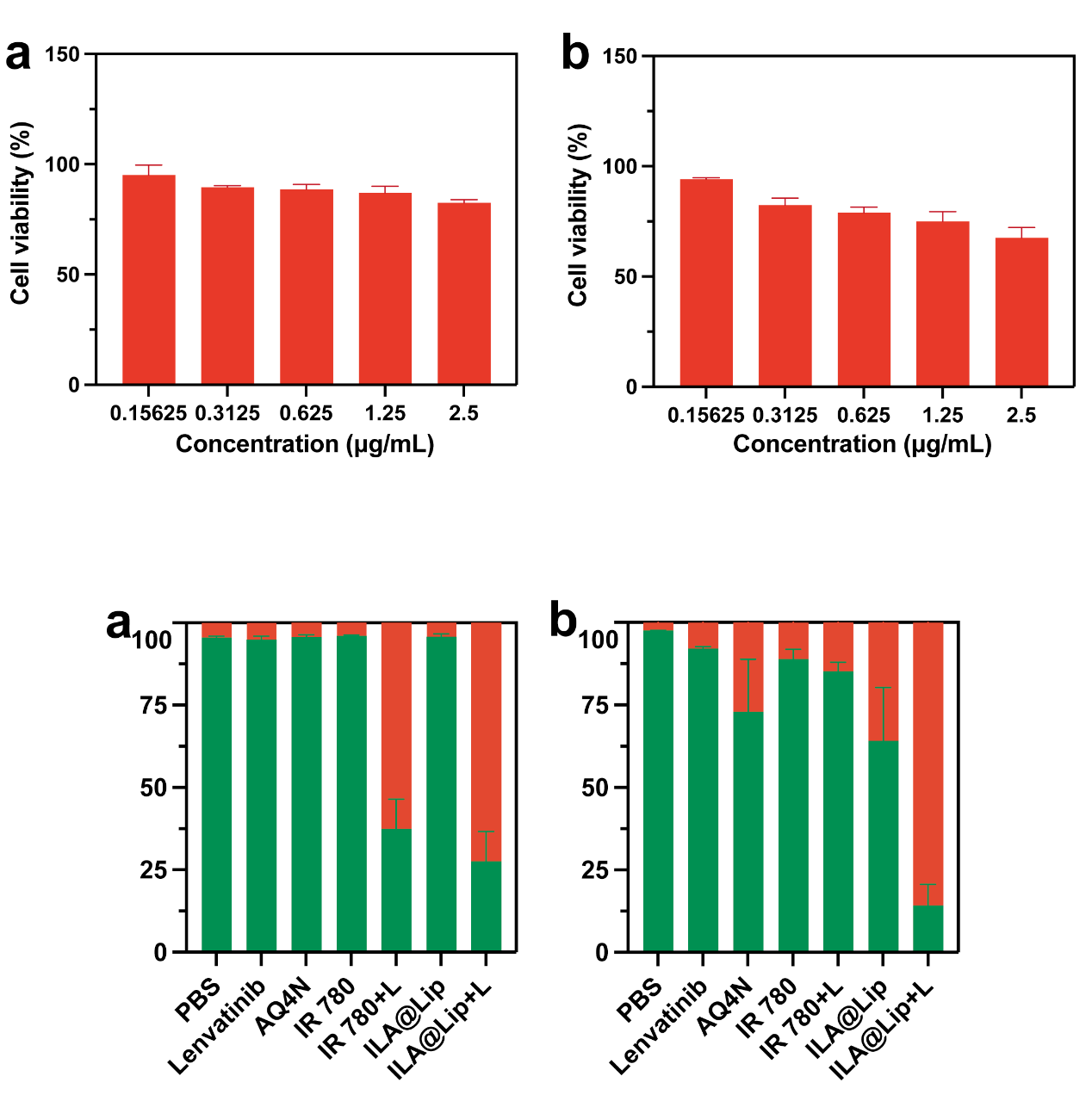


**Figure S6.** The viabilities of a) HUVEC cells and b) L929 after being treated with ILA@Lip were evaluated using CCK-8 assay. Values represent means ± SD, n= 3.

**
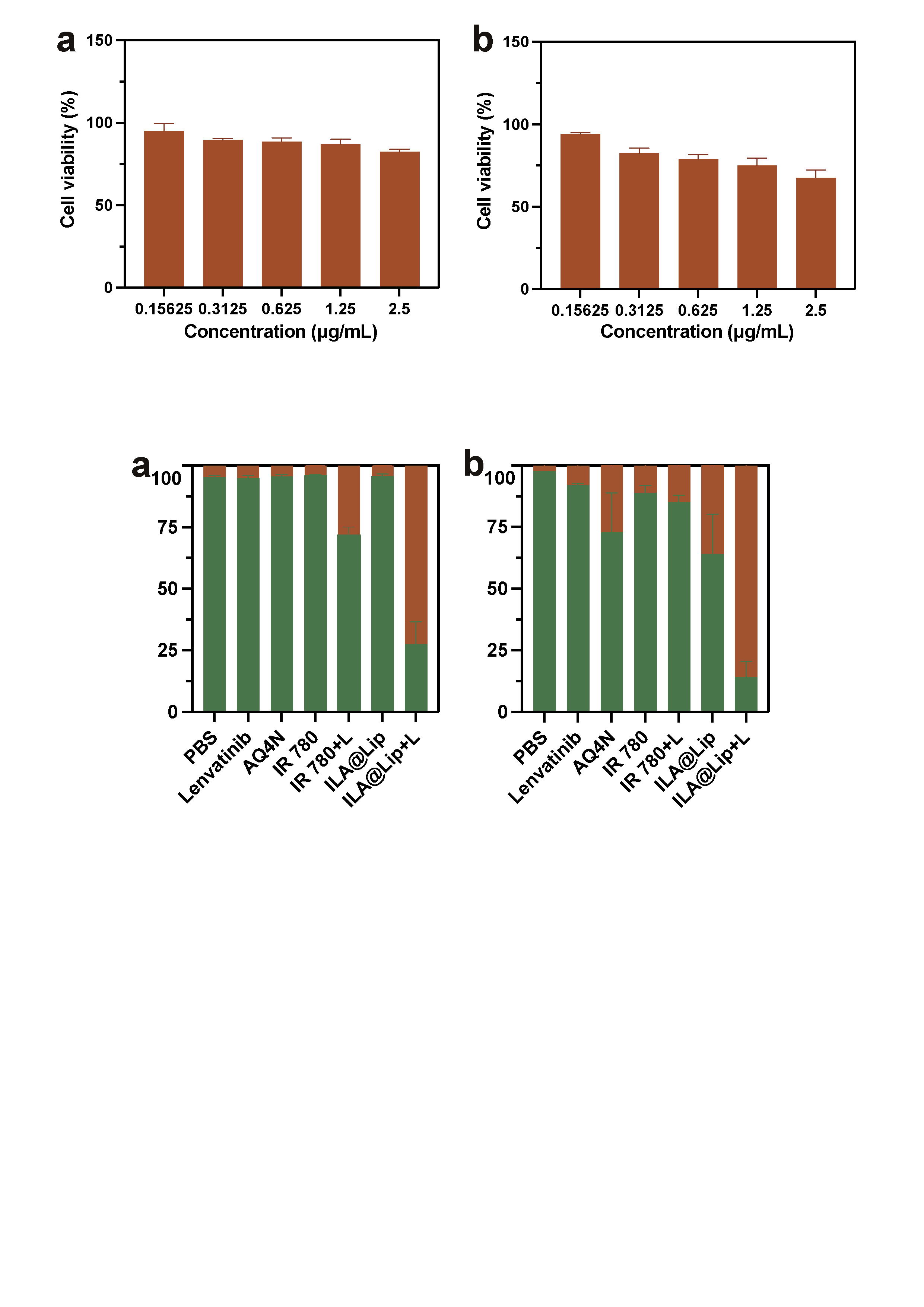
**

**Figure S7.** Quantitative analysis of Live/dead staining of MDA-MB-231 cells being treated with lenvatinib, AQ4N, IR 780, and ILA@Lip with or without laser irradiation in a) normoxia or b) hypoxia. Live cells and dead cells were signaled in green and red, respectively. (Mean ± S.D., n = 3.)

**
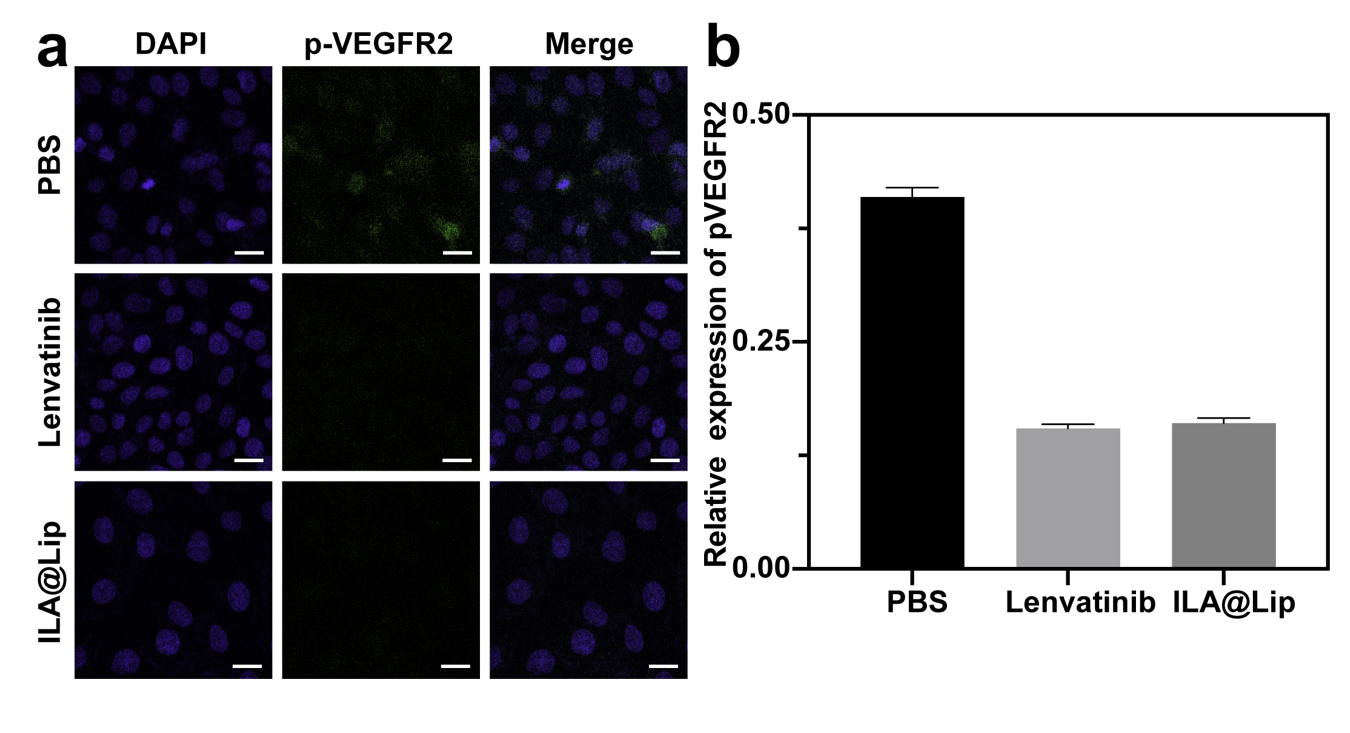
**

**Figure S8.** Lenvatinib inhibits the phosphorylation of VEGFR2 (p-VEGFR2) induced by VEGF in HUVEC. a) Immunofluorescence analysis of HUVEC cells labelling p-VEGFR2. Scale bar = 25 μm. b) Quantitative analysis of the p-VEGFR2 protein relative expression. (Mean ± S.D., n = 3.)


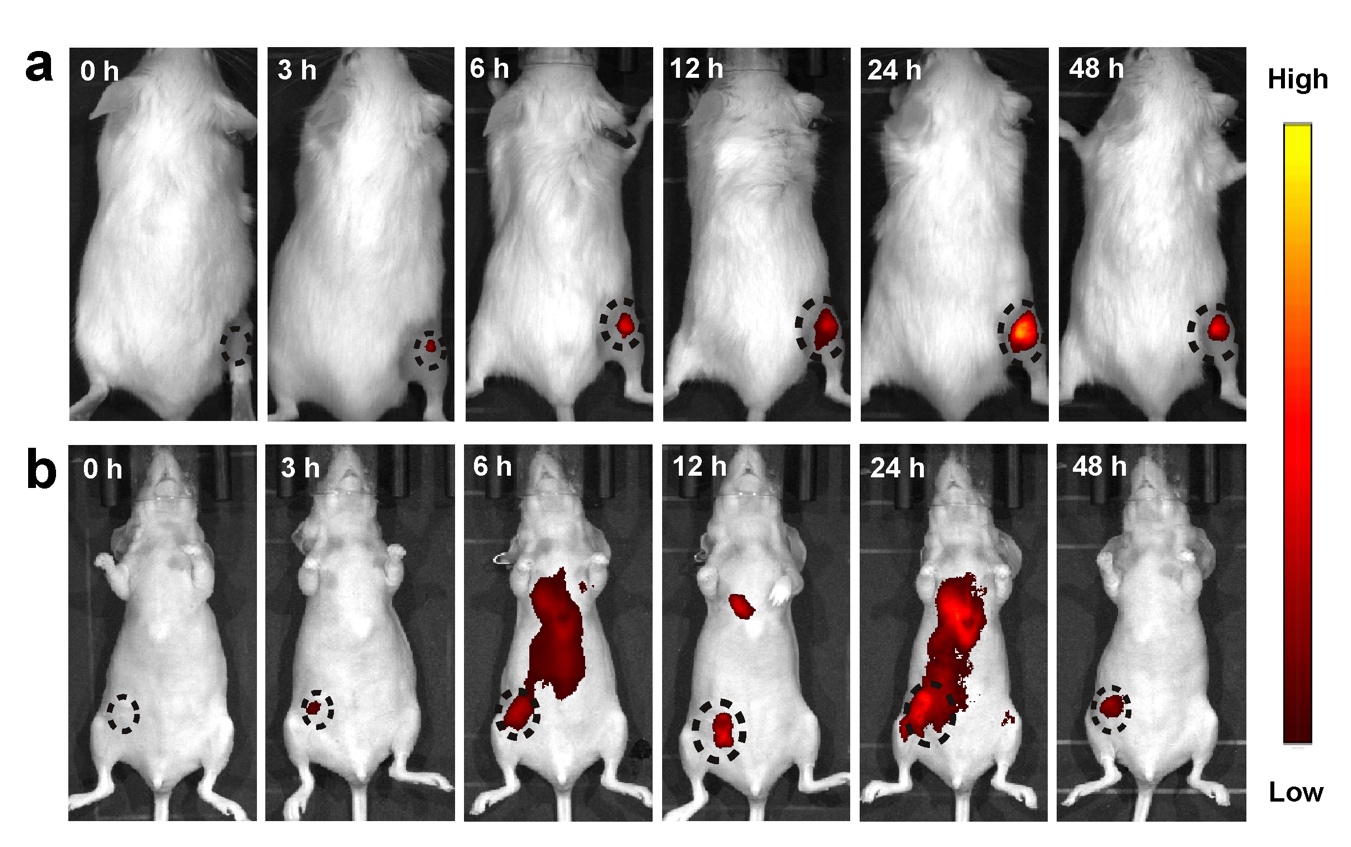


**Figure S9.** a) In vivo real-time self-monitoring of drug distribution by observing ﬂuorescence changes of IR 780 (5 mg kg^-1^) in subcutaneous 4T1 breast tumor-bearing mouse model. b) In vivo real-time self-monitoring of drug distribution by observing ﬂuorescence changes of IR 780 (5 mg kg^-1^) in orthotopic 4T1 breast tumor-bearing mouse model.


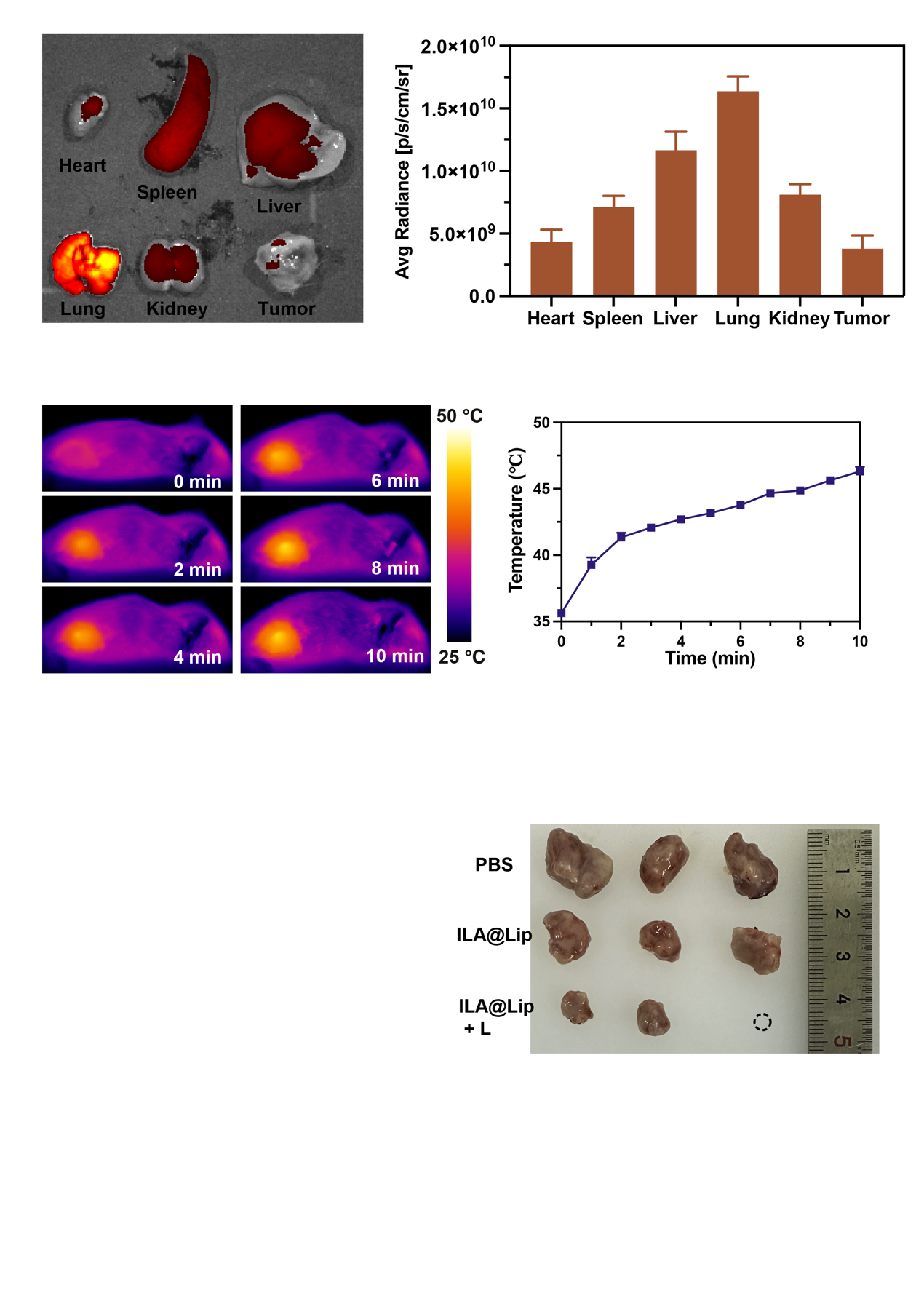


**Figure S10.** a) IR thermal images of subcutaneous 4T1 tumor-bearing mice with intravenous injection of IR 780, b) and their tumor temperature variations during a 10 min period of laser (808 nm, 1.0 W cm^-2^) irradiation at the tumor site.


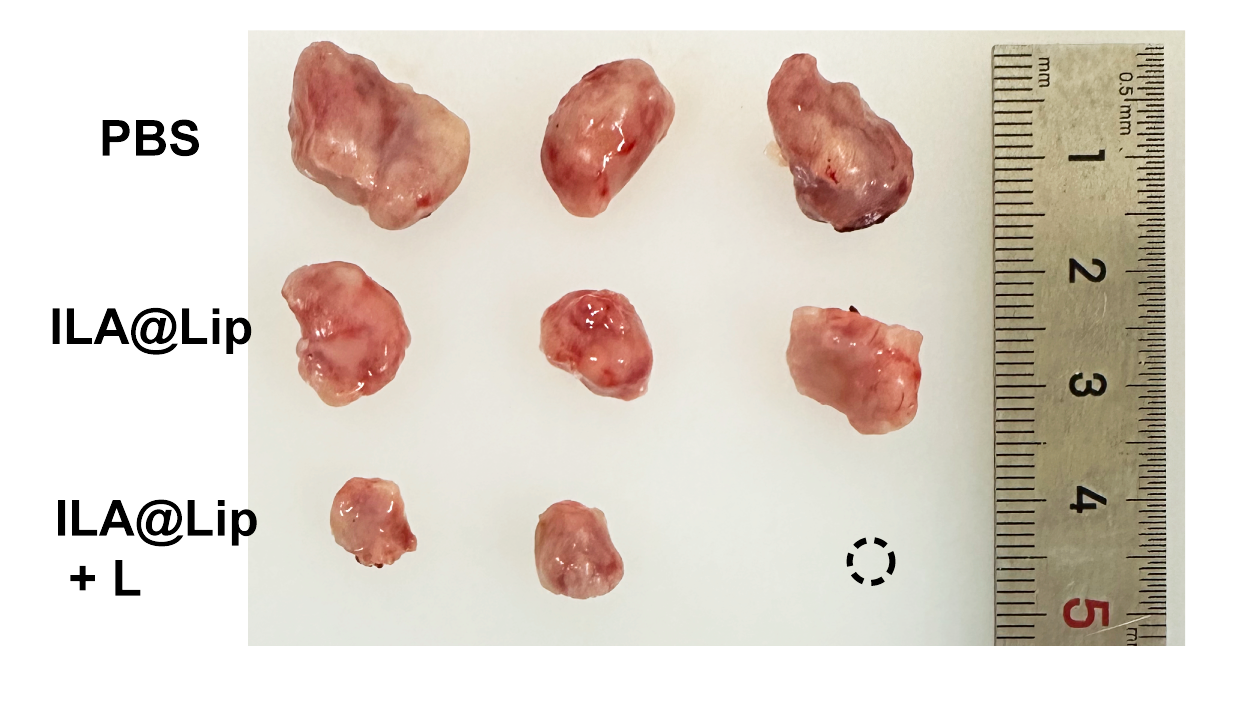


**Figure S11.** Photos of tumors from orthotopic 4T1 breast tumor-bearing mice after being treated with PBS, and ILA@Lip with or without laser irradiation (808 nm, 1.0 W cm^-2^).


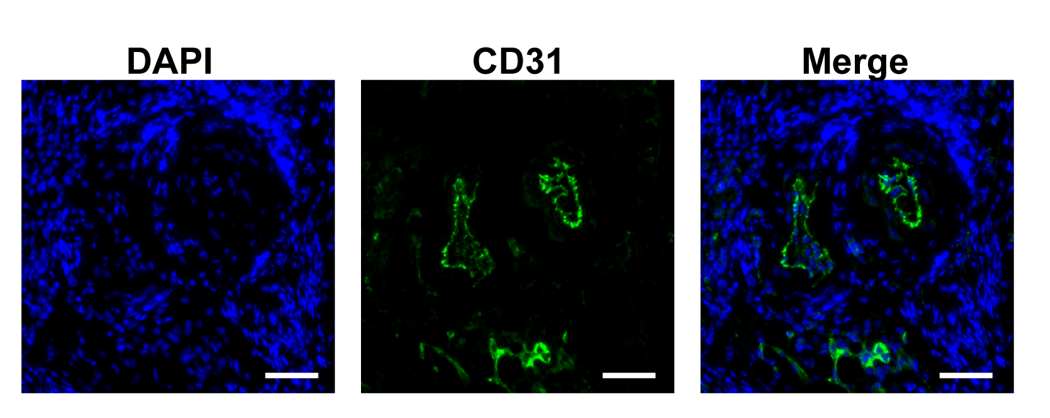


**Figure S12.** Immunoﬂuorescence images of CD31 staining in 4T1 tumors of mice with low concentration of lenvatinib. Scale bar = 50 μm.

**
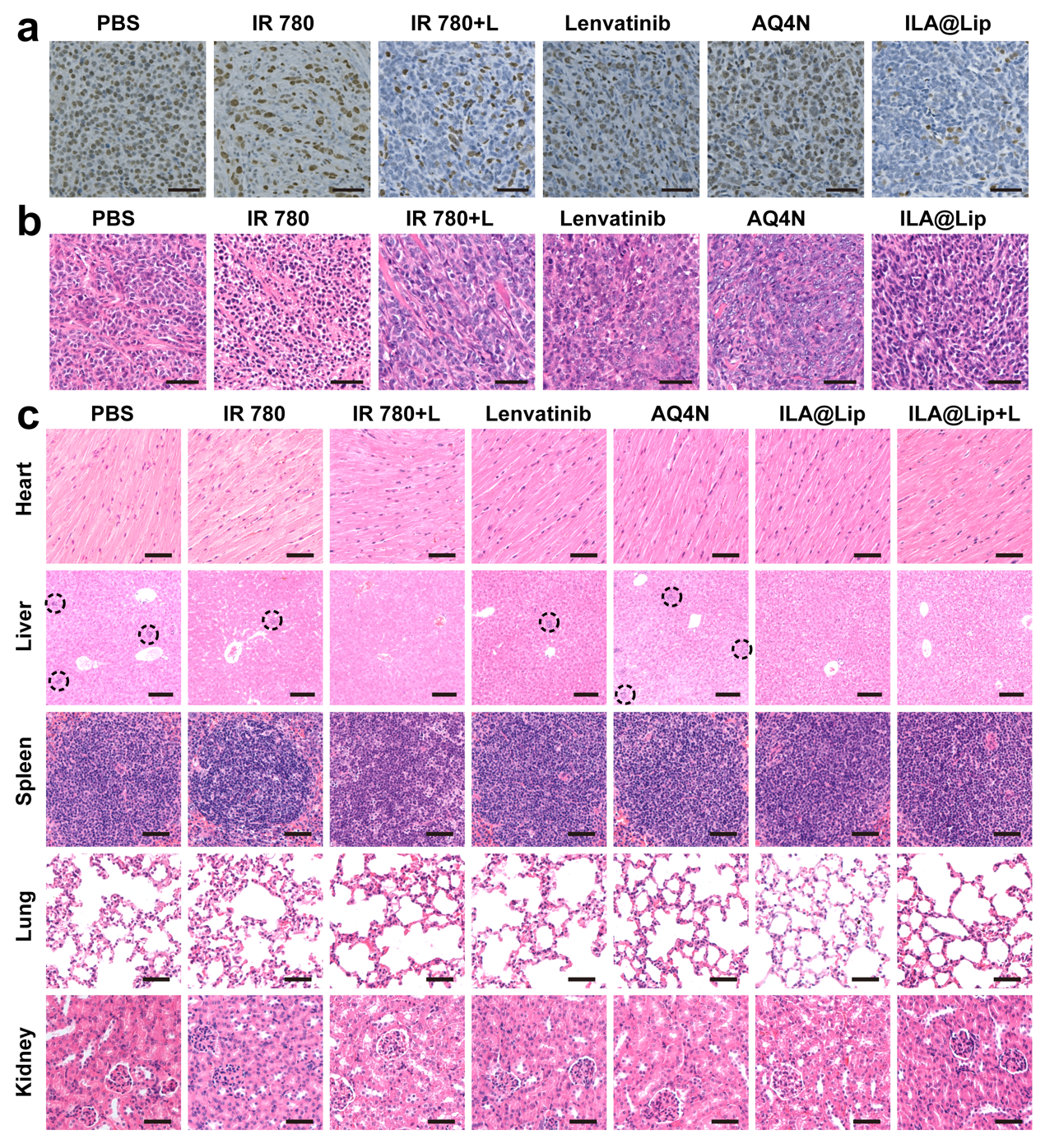
**

**Figure S13.** The immunohistochemical studies of a) Ki-67 and b) H&E of tumor cancer slices. The brown staining of the nucleus indicated the positive expression of Ki-67, and the higher the positive rate, the stronger proliferation of tumor cells. Scale bars = 50 μm. c) H&E staining of heart, liver, spleen, lung, and kidney from mice after various treatments. Circled areas are the tumor cells. Scale bars =50 μm.


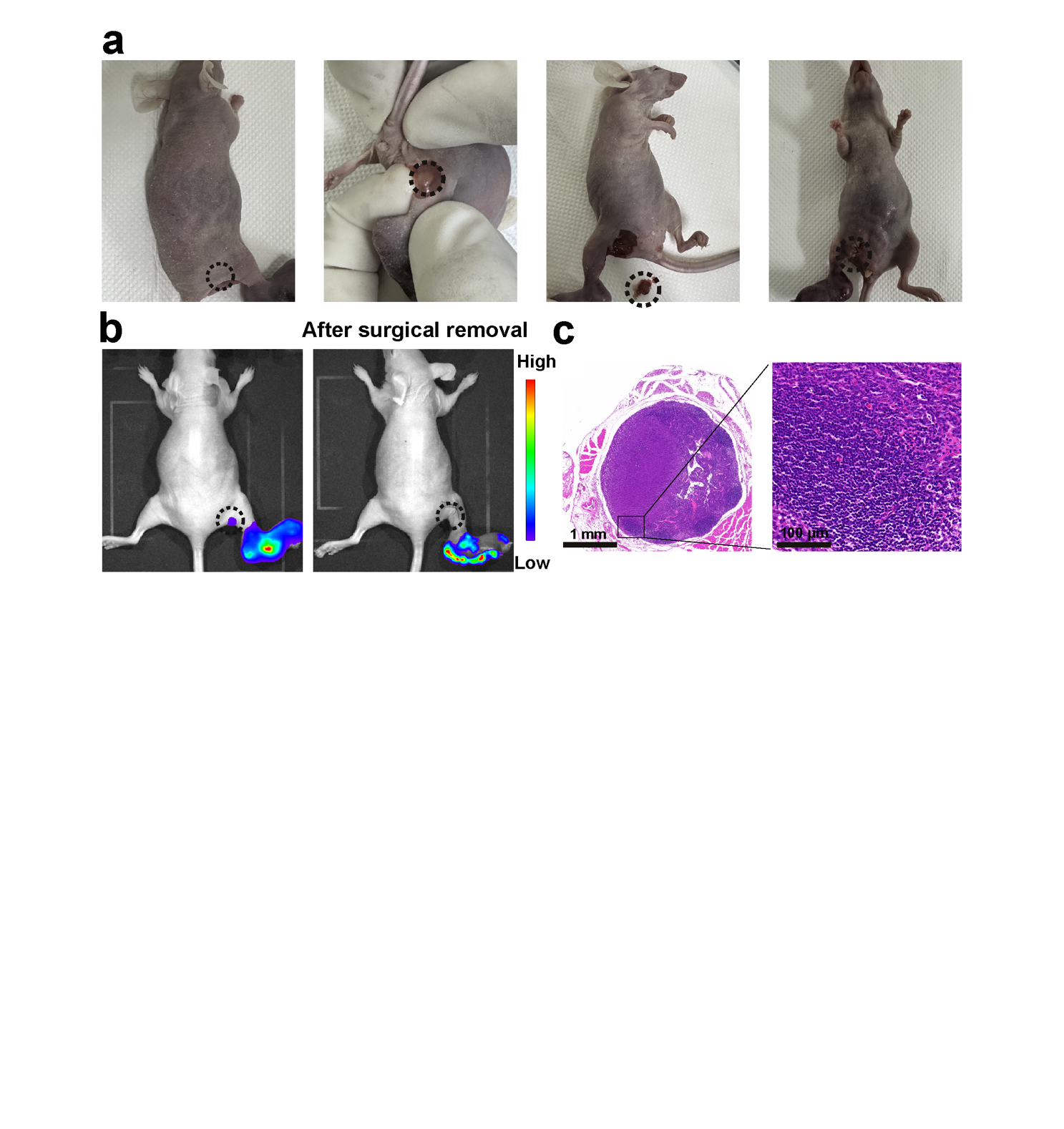


**Figure S14.** a) Photographs of a surgical procedure to remove a TNBC metastatic lymph node. b) Bioluminescence images of mice with lymph node metastasized tumors before and after surgical removal guided by NIR ﬂuorescence images. c) H&E staining of tumor slices from surgically removed TNBC metastatic lymph node.
